# Structure of ribosome-bound azole-modified peptide phazolicin rationalizes its species-specific mode of bacterial translation inhibition

**DOI:** 10.1038/s41467-019-12589-5

**Published:** 2019-10-08

**Authors:** Dmitrii Y. Travin, Zoe L. Watson, Mikhail Metelev, Fred R. Ward, Ilya A. Osterman, Irina M. Khven, Nelli F. Khabibullina, Marina Serebryakova, Peter Mergaert, Yury S. Polikanov, Jamie H. D. Cate, Konstantin Severinov

**Affiliations:** 1 Center of Life Sciences, Skolkovo Institute of Science and Technology, Moscow, Russia; 2 Institute of Gene Biology, Russian Academy of Science, Moscow, Russia; 3 Department of Chemistry, University of California, Berkeley, USA; 4 Department of Molecular and Cell Biology, University of California, Berkeley, USA; 5 Department of Chemistry, Lomonosov Moscow State University, Moscow, Russia; 6 Department of Bioengineering and Bioinformatics, Lomonosov Moscow State University, Moscow, Russia; 7 Department of Biological Sciences, University of Illinois at Chicago, Chicago, IL USA; 8 A.N. Belozersky Institute of Physico-Chemical Biology, Lomonosov Moscow State University, Moscow, Russia; 9 Institute for Integrative Biology of the Cell (I2BC), CNRS CEA University Paris-Sud, University Paris-Saclay, Gif-sur-Yvette, France; 10 Department of Pharmaceutical Sciences, University of Illinois at Chicago, Chicago, USA; 11 Waksman Institute for Microbiology, Rutgers, The State University of New Jersey, Piscataway, USA; 12 Present Address: Department of Cell and Molecular Biology, Uppsala University, Uppsala, Sweden; 13 Present Address: National Medical Research Center for Phthisiopulmology & Infectious Diseases, Moscow, Russia

**Keywords:** Peptides, Cryoelectron microscopy, Post-translational modifications, Ribosome

## Abstract

Ribosome-synthesized post-translationally modified peptides (RiPPs) represent a rapidly expanding class of natural products with various biological activities. Linear azol(in)e-containing peptides (LAPs) comprise a subclass of RiPPs that display outstanding diversity of mechanisms of action while sharing common structural features. Here, we report the discovery of a new LAP biosynthetic gene cluster in the genome of *Rhizobium* Pop5, which encodes the precursor peptide and modification machinery of phazolicin (PHZ) – an extensively modified peptide exhibiting narrow-spectrum antibacterial activity against some symbiotic bacteria of leguminous plants. The cryo-EM structure of the *Escherichia coli* 70S-PHZ complex reveals that the drug interacts with the 23S rRNA and uL4/uL22 proteins and obstructs ribosomal exit tunnel in a way that is distinct from other compounds. We show that the uL4 loop sequence determines the species-specificity of antibiotic action. PHZ expands the known diversity of LAPs and may be used in the future as biocontrol agent for agricultural needs.

## Abstract (web summary)

The authors report the identification of phazolicin (PHZ) - a prokaryotic translation inhibitory peptide - and its structure in complex with the *E. coli* ribosome, delineating PHZ’s mode of action and suggesting a basis for its bacterial species-specific activity.

## Introduction

Since the discovery of the first antibiotics, natural products of bacterial and fungal origin serve as the predominant source of new compounds with various in-high-demand functional activities in the fight against ever-growing antibiotic resistance and cancer^1,2^. While traditional activity-based screens often lead to the rediscovery of already known compounds, the ever-increasing genomic data make genome-mining-based strategies viable alternatives in the search for new bioactive compounds. Ribosomally synthesized and post-translationally modified peptides (RiPPs) comprise a diverse class of natural products that are well suited for genome-mining approaches as their biosynthetic gene clusters (BGCs) contain multiple signature genes coding for enzymes performing posttranslational modifications and the peptide precursor genes, allowing to significantly improve the quality of bioinformatics predictions^3^.

Linear azol(in)e-containing peptides (LAPs) form a subfamily of RiPPs sharing thiazol(in)e and (methyl)oxazol(in)e heterocycles, which result from cyclization of Cys and Ser (Thr) side chains during the posttranslational modification of the precursor peptide^4^. In addition to the precursor peptide-coding gene (gene A) LAP BGCs typically include genes for an YcaO-domain containing cyclodehydratase (gene D), its partner protein (gene C or gene F), FMN-dependent dehydrogenase (gene B), which oxidizes azolines to azoles, and the efflux pump^5^. Although less than two dozens of LAPs can be considered as well-characterized, they demonstrate antibacterial activity through several completely different mechanisms. For example, microcin B17 is a DNA-gyrase poison^6^, plantazolicin targets the membrane^7^, and klebsazolicin inhibits the ribosome^8^. This diversity of modes of action, unprecedented for peptides sharing common chemical features, makes LAPs a group of special interest for the search of new antibacterials.

Here, we report the discovery of a new LAP BGC in the genome of *Rhizobium* sp. Pop5, and characterization of its product, phazolicin (PHZ)—a previously unknown linear thiazole/oxazole-modified peptide, which targets the bacterial ribosome. The producing strain, a symbiotic nitrogen-fixing bacterium was isolated in a tropical forest in Los Tuxtlas (Mexico) from the root nodules of wild beans (*Phaseolus vulgaris*, hence “phazolicin”). Although the studies of natural products with antibacterial properties produced by rhizobia date back to the 1960s^9^, the number of well-characterized compounds is still very small with trifolitoxin produced by *R. leguminosarum* bv. *trifolii* T24 being the only rhizobial thiazole/oxazole-containing RiPP described to date^10^. Being active against a variety of bacteria from *Rhizobiales* including several phytopathogens, such as *Agrobacterium tumifaciens* and *A. rhizogenes*, PHZ is not only interesting because of its idiosyncratic mode of interaction with the ribosome, but also as a potential agent for biocontrol in agriculture.

## Results

### Identification of a new LAP biosynthetic gene cluster

**Fig. 1:**
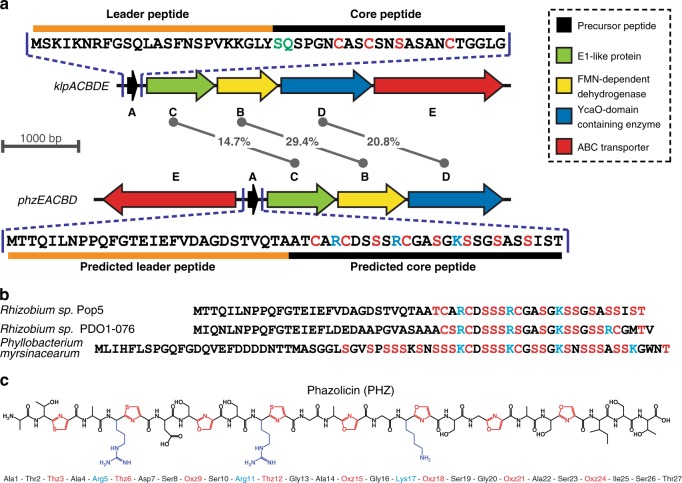
Organization of the phazolicin (PHZ) biosynthetic gene cluster. **a** Comparison of klebsazolicin (KLB) biosynthetic gene cluster (*klpACBDE*) with the cluster found in the genome of *Rhizobium* sp. Pop5. Functions of the genes forming the BGCs are listed on the right. The numbers in the middle indicate the extent of identity between the amino acid sequences of the C, B, and D gene products. The sequence of the KLB precursor peptide is shown above its BGC with the leader and core parts indicated. Residues converted to azoles in the final product are highlighted in red. Residues involved in the formation of the N-terminal amidine cycle are highlighted in green. The sequence of the precursor peptide from the newly identified BGC is shown below it. Ser and Cys residues in the sequence of PhzA precursor involved in oxazole and thiazole formation, respectively, are shown in red. Positively charged residues of the predicted core part are highlighted in blue. **b** Alignment of the amino acid sequences of precursor peptides encoded in *phzEACBD* BGC and BGCs of PHZ homologs from other *Rhizobiales* (*Rhizobium* sp. PDO1-076 and *Phyllobacterium myrsinacearum* DSM 5893). Ser, Thr, and Cys residues in the predicted core parts of the peptides are shown in red. Positively charged residues are highlighted in blue. **c** Structure of the fully modified PHZ peptide after the cleavage of the leader. Cyclized residues are shown in red (Thz and Oxz). Positively charged amino acids are highlighted in blue

Klebsazolicin (KLB) from *Klebsiella pneumoniae* is a recently characterized LAP, which inhibits prokaryotic translation through the obstruction of the ribosome exit tunnel^8^. The BGC of this RiPP (*klpACBDE*) contains genes encoding the precursor peptide (*klpA*), the three enzymes involved in the formation of azole cycles (*klpC*, *klpB*, and *klpD*), as well as a transmembrane pump (*klpE*) exporting the modified peptide. We used the amino acid sequences of KlpB and KlpD proteins as queries to search the NCBI non-redundant protein database using the PSI-BLASTP algorithm^11^ followed by manual analysis of the genomic environment of the identified gene orthologues. Along with several previously described clusters of KLB homologs^8^ a distinct BGC *phzEACBD* was found in the genome of *Rhizobium* sp. Pop5. Two other related BGCs were found in the genomes of *Rhizobium* sp. PDO1-076 and *Phyllobacterium myrsinacearum* DSM 5893, also belonging to the order *Rhizobiales*. The alignment of amino acid sequences of the predicted core parts of precursor peptides revealed a similar pattern of Cys/Ser/Thr residues, which potentially could be cyclized in the mature LAP product (Fig. 1b, red). The putative PhzA precursor peptide has an amino acid sequence significantly different from that of the KLB precursor KlpA (Fig. 1a). As in the case with KlpA, the C-terminal part of the predicted PhzA precursor peptide is enriched in Ser, Thr, and Cys residues but also contains several positively charged residues absent in the core part of KlpA. Moreover, PhzA and its relatives lack the X-X-(S/T)P motif required for the formation of the N-terminal amidine cycle, which is strictly required for the activity of KLB^12^. Therefore, we hypothesized that the *phzEACBD* and similar BGCs from other *Rhizobiales* might encode closely related LAPs with structural and functional properties different from those of KLB or other known compounds of the family.

### Purification and characterization of a new LAP

Reverse-phase HPLC analysis of medium after cultivation of the *Rhizobium* sp. Pop5 led to the identification of several fractions absorbing at 254 nm, a wavelength previously used in the purification of other azole-containing compounds (Supplementary Fig. 1A)^8,13^. MALDI-TOF-MS analysis of the selected fractions showed the presence of compounds, which, based on the observed masses, appeared to be the products of the PhzA precursor peptide posttranslational modification (Supplementary Fig. 1B). The major compound with monoisotopic MH^+^ 2363.9 was named PHZ (Supplementary Fig. 1C). This mass matches the mass of the C-terminal part of the PhzA precursor peptide, if it was cleaved between the two alanine residues […Thr-Ala-↓-Ala-Thr-Cys…] and contained eight azole rings (loss of 20 Da per installation of each cycle corresponds to −18 Da for each dehydration/cyclization reaction and −2 Da for each azoline oxidation event) (Fig. 1c). Two other major compounds detected in the growth media (Supplementary Fig. 1B) also contained eight azole cycles but had an alternative site for the leader peptide cleavage situated one or two residues closer to the N-terminus of the precursor. These compounds are further referred as A-PHZ (MH^+^ = 2434.9) and TA-PHZ (MH^+^ = 2536.0). High-resolution MS measurements for PHZ, A-PHZ, and TA-PHZ resulted in MW values of 2362.8679, 2433.9052, and 2534.9524, which are all within 1.5 ppm of the corresponding masses calculated based on the brutto-formulas (Supplementary Fig. 2). Although due to extensive modification the fragmentation of the PHZ was relatively poor, MS/MS analysis using both ESI-MS/MS and MALDI-MS/MS techniques allowed predictions of the positions of azole cycles in the structure of PHZ (Supplementary Fig. 3). PHZ is a 27-residue long peptide with three thiazole and five oxazole cycles in its structure. The cycles are distributed evenly across the core part of the precursor peptide with every third residue being turned into an azole (Fig. 1c). In addition to the major fractions, several minor forms with a fewer (5–7) number of azole cycles were detected in the cultivation medium (Supplementary Fig. 1B). Compounds with less than eight cycles lacked the C-terminal cycles, consistent with the N- to C-terminus direction of modification of the PHZ precursor peptide.

### PHZ demonstrates antimicrobial activity against Rhizobiales

When the purified PHZ was tested against *Escherichia coli* BW25113 grown on rich solid medium, small growth inhibition zones were observed only when the compound was applied in high concentrations (5–10 mM). Many known RiPPs demonstrate a narrow spectrum of antibacterial activity and are usually active against bacteria, which are evolutionary close to the producing strain^7,14,15^ serving as powerful weapons in the competition for the ecological niche between closely related species. Accordingly, we tested PHZ against a panel of microorganisms from the order *Rhizobiales* (class *Alphaproteobacteria*) grown in a rich liquid medium. The compound proved to be highly active against bacteria from genera *Rhizobium*, *Sinorhizobium* (*Ensifer*), and *Azorhizobium* with the minimal inhibitory concentrations (MICs) being around 1 μM (Table 1). PHZ is less active against two tested strains of *Agrobacterium*, while microorganisms from the genus *Mesorhizobium* appeared to be PHZ-resistant (Table 1). PHZ was also tested against a small set of plant pathogens, plant-associated and soil microorganisms from *Gammaproteobacteria* (*Erwinia amylovora*, *Pantoea ananatis* PA4*, Pseudomonas putida* KT2440, *P. fluorescens* Pf-5), *Firmicutes* (*Bacillus subtilis* 168, *B. cereus* ATCC 4342), and *Actinobacteria* (*Arthrobacter* sp. ATCC21022, *Microtetraspora glauca* NRRL B-3735). However, no activity was observed. Taken together, these observations demonstrate that, similarly to a number of other LAPs, PHZ is a narrow-spectrum antimicrobial compound specifically targeting several genera from *Rhizobiales*.

**Table 1:** Phazolicin MICs for various bacterial strains in rich YEB medium and *Rhizobium* medium (RM)

Microorganism (Strain)	Medium	MIC (µM)	MIC (µg/ml)
*Rhizobium leguminosarum* bv. *viciae* 3841	RM	1	2.4
*Rhizobium leguminosarum* bv. *phaseoli* 4292	RM	0.125	0.3
*Rhizobium leguminosarum* bv. *phaseoli* RCR 3622	RM	2	4.7
*Rhizobium etli* DSM 11541	RM	0.25	0.6
*Rhizobium tibethicum* DSM 21102	RM	1	2.4
*Agrobacterium tumefaciens* C58C1	YEB	64	151.3
*Agrobacterium rhizogenes* ARQUA1	YEB	64	151.3
*Mesorhizobium ciceri*	YEB	>128	>302.6
*Mesorhizobum loti* MAFF303099	YEB	>128	>302.6
*Mesorhizobium thianshanense* HAMBI 3372	YEB	>128	>302.6
*Azorhizobium caulinodans* ORS571	YEB	2	4.7
*Sinorhizobium fredii* HH103	YEB	0.25	0.6
*Sinorhizobium medicae* WSM419	YEB	4	9.5
*Sinorhizobium meliloti* Sm1021	YEB	1	2.4
*Sinorhizobium meliloti* Sm1021 pSRK	YEB	1	2.4
*Sinorhizobium meliloti* Sm1021 pSRK-*phzE*	YEB	>128	>302.6

The resistance of producing strains to RiPPs with antimicrobial activity often results from the efficient export of the mature compound by a specific transporter encoded in the corresponding BGC^16^. However, there are examples of BGCs containing additional genes providing self-immunity through different mechanisms^17,18^. Expression of a plasmid-borne copy of the *phzE* gene, which was annotated as an exporter pump, in *S. meliloti* Sm1021 conferred resistance to external PHZ and increased the MIC more than 100-fold in comparison with the strain carrying an empty vector (λ). These data confirm the role of PhzE transporter in self-immunity of the producing strain to PHZ.

### PHZ inhibits bacterial protein translation in vivo and in vitro

**Fig. 2:**
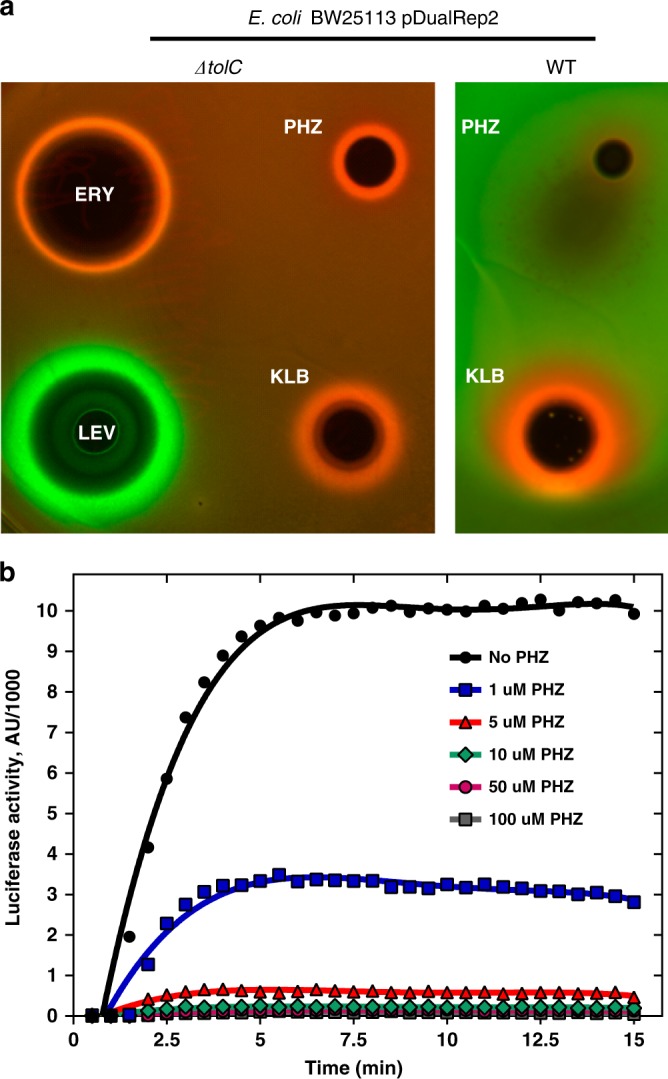
Phazolicin is an inhibitor of protein synthesis both in vivo and in vitro. **a** In vivo testing of PHZ activity using BW25113 Δ*tolC* pDualRep2 and BW25113 pDualRep2 (WT) reporter strains. The induction of RFP expression (green halo around the inhibition zone, pseudocolors) is triggered by DNA-damage while the induction of Katushka2S protein (red halo) occurs in response to ribosome stalling. Erythromycin (ERY), levofloxacin (LEV), and klebsazolicin (KLB) are used as controls. **b** Kinetic curves showing inhibition of protein synthesis by increasing concentrations of PHZ in the in vitro cell-free translation in *E. coli* S30 extract. AU arbitrary units

To determine the mode of action of PHZ we tested its activity in vivo using an *E. coli*-based double-reporter system that allows one to identify antibiotics inducing ribosome stalling or inhibiting DNA replication^19^. In this system, pDualrep2 plasmid harbors two genes encoding fluorescent proteins under different regulation: the red fluorescent protein (RFP) gene is transcribed in the presence of sublethal concentrations of SOS-response inducing agents (e.g., fluoroquinolones or microcin B17), while the gene for far-red protein Katushka2S is transcribed only when translation inhibitors (e.g., macrolides) are present in the medium in sublethal concentrations. Thus, when a drop containing antimicrobial agent is deposited onto the lawn of cells carrying pDualrep2, the compound diffuses into the media and usually kills cells adjacent to the deposition spot. However, further away from the deposition spot at the outer edge of a growth inhibition zone the compound is present in a sublethal concentration and can induce production of either the RFP or Katushka2S fluorescent protein depending on whether the compound targets DNA replication or translation, respectively. Similar to KLB and erythromycin (ERY), PHZ induces expression of Katushka2S but not RFP, indicating that PHZ is an inhibitor of translation (Fig. 2a, left panel). In this assay, we observed a difference in the size of PHZ-induced inhibition zones between the *E. coli* strain with the deleted *tolC* gene and the wild-type strain (Fig. 2a, right panel). TolC is a major outer membrane multidrug efflux porin that is responsible for the export of various compounds from the cell including siderophores (e.g., enterobactin^20^), macrolide antibiotics^21^, and some RiPPs (e.g., microcin J25^22^). In contrast to the situation observed with PHZ, the KLB-induced inhibition zones were comparable in size between the Δ*tolC* and the wild-type *E. coli* strains, suggesting that PHZ but not KLB is subject to TolC-dependent efflux.

To further confirm that PHZ is a protein synthesis inhibitor, we tested its ability to decrease the translation of the luciferase mRNA in vitro using an *E. coli* S30 extract. Concentration-dependent inhibition of in vitro translation by PHZ was observed (Fig. 2b) with 1 μM of PHZ decreasing the luciferase signal by approximately three-fold (Fig. 2b, blue curve), while at 5 μM PHZ inhibited translation by ~95% (Fig. 2b, red curve).

### PHZ obstructs the nascent peptide exit tunnel (NPET) of the ribosome

Protein synthesis inhibitors can interfere with the activity of the ribosome or any of the various other enzymes associated with protein production (translation factors, aminoacyl-tRNA synthetases, etc.). Following the analogy with KLB, we proposed that PHZ acts upon the bacterial ribosome. To determine the exact mechanism of translation inhibition by PHZ we attempted to crystallize the 70S ribosome from *Thermus thermophilus (Tth)* in complex with PHZ, mRNA, and tRNAs, as previously done with KLB^8^, and several other translation inhibitors^23–30^. Despite numerous attempts, we were not able to observe any positive electron density peaks corresponding to the ribosome-bound PHZ anywhere within the *Tth* 70S ribosome. Moreover, in vitro translation inhibition experiments using *E. coli (Eco)* 70S or hybrid 70S (*Eco* 30S subunit + *Tth* 50S subunit) demonstrated that PHZ does not target *Tth* ribosome (Supplementary Fig. 4). However, the same data also suggested that PHZ does target the large ribosomal subunit of the *E. coli* ribosome.

**Fig. 3:**
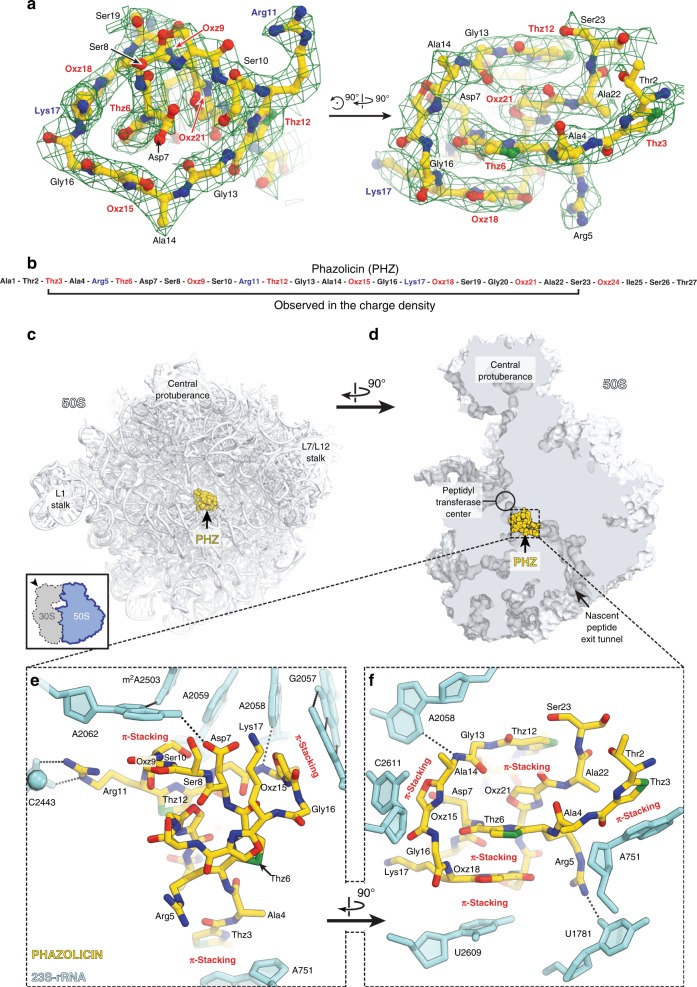
The structure of PHZ in complex with the bacterial ribosome. **a** Cryo-EM map of PHZ in complex with the *E. coli* 70S ribosome (green mesh). The fitted model of the compound is displayed in its respective charge density viewed from two different perspectives. The map is contoured at 2.5*σ*. Carbon atoms are colored yellow, nitrogen atoms are blue, oxygen atoms are red, and sulfur atoms are green. **b** Schematic diagram showing that only residues 2–23 of the ribosome-bound PHZ molecule are visible in the cryo-EM map. **c**, **d** Overview of the PHZ-binding site (yellow) on the *E. coli* large ribosomal subunit (light blue) viewed from two different perspectives. In **c**, the 50S subunit is viewed from the inter-subunit interface (30S subunit is removed for clarity) as indicated by the inset. The view in **d** is from the cytoplasm onto the A site. **e**, **f** Close-up views of the PHZ-binding site in the ribosome exit tunnel. *E. coli* numbering of the nucleotides in the 23S rRNA is used. Potential H-bond interactions are indicated with dotted lines

Since the crystallization-based approach was unsuccessful, we switched to cryo-EM to determine the structure of PHZ bound to the sensitive *Eco* 70S ribosome. The obtained charge density map, characterized by the overall resolution of 2.87 Å according to the “gold-standard” Fourier shell correlation (FSC) method (Supplementary Fig. 5A), revealed PHZ bound to the ribosome (Fig. 3a). The high resolution and excellent quality of the map in the PHZ-binding site (Supplementary Fig. 5B), allowed us not only to fit its atomic model (residues 2–23; Fig. 3b) but also to confirm the positions of seven azoles in the structure of the modified peptide proposed according to the MS–MS analysis. While the results of tandem mass spectrometry indicate another oxazole present at the position 24, we left Ser23 unmodified in the model since Ser24 was not visible in the map due to its poor quality in that region (Fig. 3a).

Similar to KLB, linear modified PHZ peptide forms a compact globule that binds in the upper part of the NPET of the 50S ribosomal subunit where it extensively interacts with the 23S rRNA and ribosomal proteins (Fig. 3c, d; Supplementary Movie 1). In comparison to antibiotics of smaller molecular weight, such as ERY, which binds at the same location but only partially occludes the NPET (Supplementary Fig. 6A, B), binding of both KLB and PHZ results in nearly complete obstruction of the NPET (Supplementary Fig. 6C, D). A prominent feature of the ribosome-bound PHZ molecule is its complex intramolecular interactions that involves both face-to-face and edge-to-face π–π stacking of Thz12, Oxz21, Thz6, and Oxz18, along with the nucleobase U2609 from the 23S rRNA (Fig. 3f; Supplementary Fig. 7D). Moreover, we observed two major types of interactions between the PHZ and the nucleotides of the 23S rRNA. First, three azoles (Thz3, Oxz15, and Oxz18) are involved in π–π stacking with nucleotides A751, C2611, and U2609, respectively (Fig. 3e, f). Second, two positively charged side chains of Arg5 and Arg11, as well as hydrophilic side chains of Asp7 and Ser8, form additional stabilizing hydrogen bonds (H-bonds) with nucleobases and backbone phosphate groups of the 23S RNA (Fig. 3e, f; Supplementary Fig. 7A–C). The presence of intramolecular π–π stacking and positively charged side chains, as well as the absence of the N-terminal amidine cycle, make the mode of PHZ binding significantly different from that of KLB. Unlike KLB, the N-terminal part of PHZ is not critical for binding to the ribosome, which is further supported by the observation that naturally occurring longer forms with an alternative leader cleavage site (A-PHZ and TA-PHZ) demonstrate the same level of in vitro translation inhibition activity as PHZ (Supplementary Fig. 8).

**Fig. 4:**
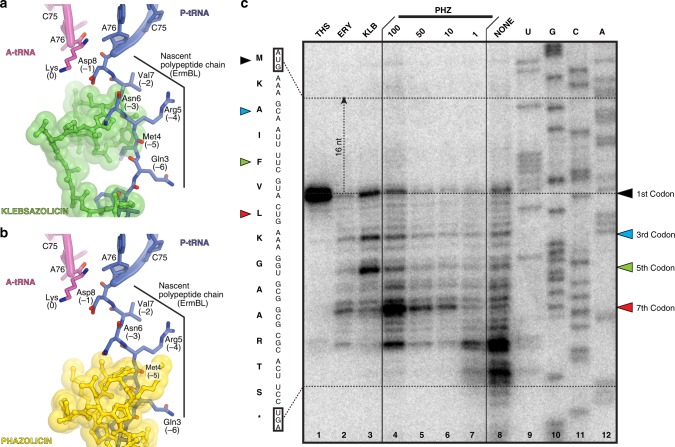
PHZ inhibits the elongation step during protein synthesis. **a**, **b** In silico modeling of the nascent polypeptide chain in the ribosome exit tunnel in the presence of klebsazolicin (**a**, PDB entry 5W4K^8^) or phazolicin (**b**, current structure) using the cryo-EM structure of the 70S ribosome with ErmBL peptide bound to the P-site tRNA (PDB entry 5JTE^31^). **c** Ribosome stalling by PHZ on *trpL-*2Ala mRNA in comparison with other translation inhibitors (thiostrepton, THS; erythromycin, ERY; klebsazolicin, KLB), as revealed by reverse-transcription primer-extension inhibition (toe-printing) assay in a cell-free translation system. *trpL-*2Ala mRNA nucleotide sequence and the corresponding amino acid sequence are shown on the left. Colored triangles show ribosome stalling at 1st, 3rd, 5th, and 7th codons. Note that owing to the large size of the ribosome, the reverse transcriptase stops at the nucleotide + 16 relative to the codon located in the P-site

Interestingly, the PHZ-binding site overlaps with those of several other classes of antibiotics, such as macrolides and type B streptogramins. Comparison of the structures of ribosome-bound PHZ and KLB reveals that PHZ binds further away than KLB from the peptidyl transferase center (PTC) of the ribosome. We superimposed our new structure of the ribosome-bound PHZ (Fig. 4a) or the previously published KLB (Fig. 4b) with the cryo-EM structure of the 70S ribosome containing ErmBL nascent peptide chain connected to the P-site tRNA^31^. Apparently, there is more space between the ribosome-bound drug and the PTC in the case of PHZ in comparison to KLB. This suggests that PHZ should, in principle, allow for a few more amino acids to be incorporated into the nascent polypeptide chain before it encounters the NPET-bound PHZ molecule and translation stalls. To test this hypothesis, we performed a toe-printing (primer-extension inhibition) assay, which allows identification of drug-induced ribosome stalling on a given mRNA template with a single-nucleotide precision^32^. Because PHZ causes strong induction (stalling of the ribosomes) on Dualrep2 reporter used in our in vivo bioactivity test (Fig. 2a), we have chosen the corresponding mRNA (*trpL*-2Ala) as a template for our toe-printing assay. Addition of PHZ to the PURExpress cell-free transcription–translation system programmed with *trpL*-2Ala mRNA resulted in dose-dependent ribosome stalling at the seventh codon of mRNA (Fig. 4c, lanes 4–7), while the addition of KLB induced major stalling at the third and the fifth codons (Fig. 4c, lane 3) corroborating our hypothesis. Moreover, these data indicate that PHZ is an inhibitor of the elongation step during protein synthesis, as is the KLB. In the absence of any inhibitors in our toe-printing reactions, ribosomes stall at codons 9–10 due to a specific secondary structure in the mRNA (Fig. 4c, lane 8).

### Species-specificity of PHZ action

**Fig. 5:**
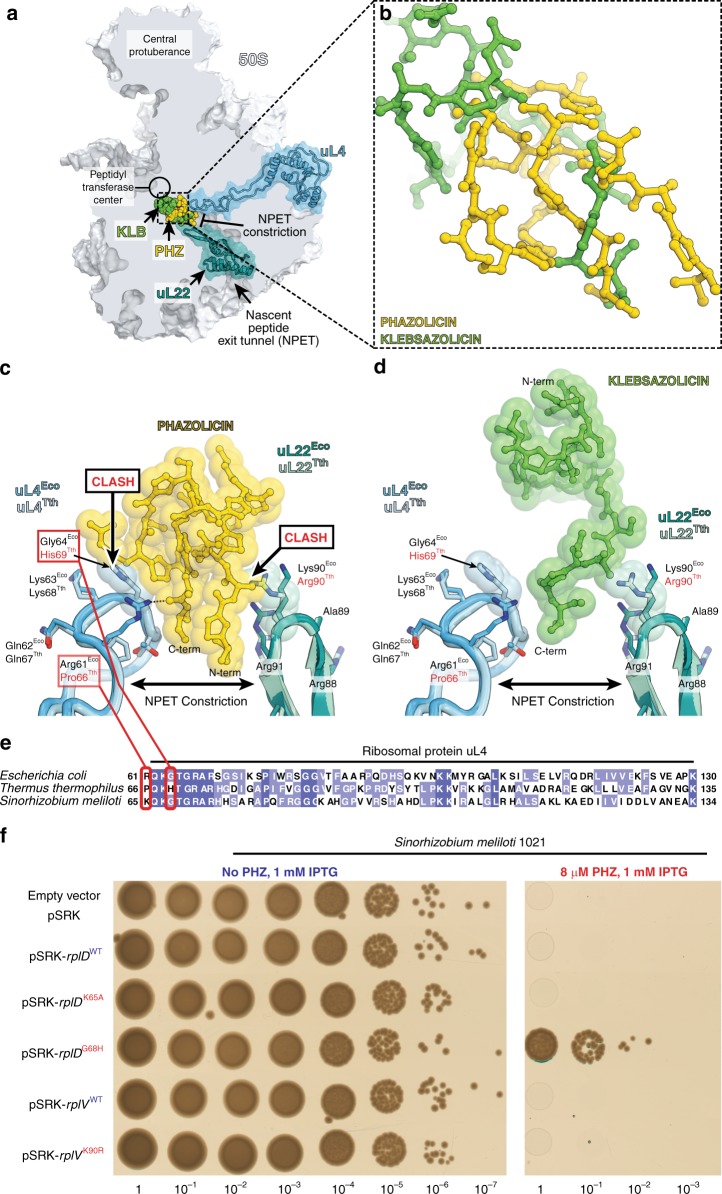
The loop of ribosomal protein uL4 defines species-specificity of PHZ action. **a** Comparison of PHZ (yellow) and KLB (green) binding sites in the ribosome exit tunnel. The 50S subunit is shown in light blue. Ribosomal proteins uL4 and uL22 are highlighted in blue and teal, respectively. **b** Superposition of the ribosome structures with bound KLB (PDB entry 5W4K^8^) and PHZ based on the alignment of the 23S rRNAs. Ribosome parts are omitted for clarity. Note that KLB binds closer to the PTC than PHZ. **c**, **d** Close-up view of PHZ **c** and KLB **d** interactions with the loops of proteins uL4 and uL22 forming the NPET constriction site. Superposition of the loops of proteins from *E. coli* and *T. thermophilus* is shown. Note that sterical clashes occur only with the side chains in the loops of proteins uL4/uL22 from *T. thermophilus*. **e** Multiple sequence alignment of the homologous parts of the ribosomal protein uL4 from several bacterial species. Red boxes highlight the amino acid residues of uL4, which either clash with the PHZ (His69 in *Tth*) or interact with it in our structure (Arg61 in *Eco*). **f** Growth of *S. meliloti* Sm1021 and its derivatives expressing plasmid-borne copies of WT or mutant rplD or rplV genes (encoding ribosomal proteins uL4 and uL22, respectively) on agar plates without PHZ (left panel) or with 8 μM PHZ (right panel). Note that only overexpression of Tth-like version of uL4 protein in S. *meliloti* confers resistance to PHZ

The PHZ-binding site is adjacent to the NPET constriction point—the most narrow part of the NPET formed by the extended loops of the ribosomal proteins uL4 and uL22 (Fig. 5a, b). The N-terminal and C-terminal parts of the PHZ molecule are located between the uL4 and uL22 loops (Fig. 5c) and occupy more space in this part of the NPET than the C-terminal part of KLB (Fig. 5d). The amino acid sequences of uL4 and uL22 proteins including their loop regions vary between distinct bacteria (Fig. 5e). Superpositioning of our *Eco* 70S ribosome structure with bound PHZ with the structure of *Tth* 70S reveals that residues His69 of the *Tth* protein uL4 sterically clashes with the PHZ molecule (Fig. 5c) rationalizing why PHZ does not bind to or act upon the *Tth* ribosome. Because the same residue in *Eco* ribosome is represented by a less bulky Gly64 (uL4), we hypothesized that the differences in the uL4 loop sequence are responsible for the inability of PHZ to bind in the NPET and, hence, to inhibit the translation by *Tth* ribosome. To test our hypothesis, we expressed a plasmid-borne copy of the *S. meliloti rplD* gene encoding for the uL4 protein carrying the *Tth*-like G68H substitution (*rplD*^G68H^) and showed that the loop structure of *Tth* uL4 confers resistance to PHZ and allows growth of otherwise sensitive strain on the medium with 8xMIC of the antibiotic (Fig. 5f). The expression of rplD^K65A^ mutant, which carries an alanine substitution of the *Eco* Arg61-equivalent (Fig. 5e) that forms H-bond with the PHZ molecule in our structure (Fig. 5c), does not confer resistance to PHZ. This is likely due to multiple contacts, which the PHZ molecule forms with the ribosome, and the loss of one H-bond does not affect the overall binding. The same superpositioning of the *Eco* and *Tth* ribosome structures also revealed that residue Arg90 of the *Tth* protein uL22 sterically clashes with the PHZ molecule (Fig. 5c), while the comparable in size-equivalent residue Lys90 in the *Eco* protein uL22 does not clash with the PHZ and is repositioned to the side relative to the ribosome-bound PHZ to avoid a similar clash. Unlike His69^Tth^, which is located in a confined pocket and cannot re-adjust its position in response to PHZ binding, residues Arg90^Tth^ and Lys90^Eco^ have more space around and should be able to easily readjust their positions when PHZ is present to avoid a possible clash. Indeed, the substitution of Lys90 in the loop of protein uL22 with a larger Arg residue (rplV^K90R^) does not render any resistance to PHZ (Fig. 5f). Thus, the fine structure of the PHZ-binding site (formed in part by the loop regions of ribosomal proteins uL4 and uL22) together with the repertoire of peptide transporters involved in the uptake contribute to the specificity of the antibiotic action.

## Discussion

The newly discovered antibiotic PHZ represents the second known example of a linear azole-containing peptide with a known mechanism of action and mode of binding to its intracellular target, the ribosome. Although the PHZ and the previously described LAP klebsazolicin both inhibit the elongation step of prokaryotic translation through the obstruction of the NPET of the large ribosomal subunit, the mode of binding of PHZ to the bacterial ribosome is significantly different from that of KLB. Unlike KLB, which does not appear to have extensive intramolecular interactions, four out of eight azole rings of PHZ form continuous π–π-stacking system (Supplementary Fig. 5D), which apparently confers the rigidity and stability of the antibiotic in its binding site. Moreover, PHZ contains several positively charged side chains, which provide additional anchoring on the ribosome through the electrostatic interactions with the negatively charged 23S rRNA (Fig. 3e), and which were absent in KLB. Finally, PHZ appears to be the first ribosome-targeting antibiotic with a pronounced bacterial species-specific mode of action, which is directly related to its mode of interaction with the molecular target. PHZ can bind to the ribosomes and act upon those species whose ribosomal protein uL4 contains glycine (or non-bulky side chain amino acid residues) in the position equivalent to Gly64 in *E. coli* that is located at the tip of the extended loop of this protein. The residue equivalent to Gly64 of the *E. coli* protein uL4 is not conserved in other bacterial species. The majority of bacteria (including *E. coli*) possess glycine at this position (Fig. 5e). However, others (e.g., *T. thermophilus*) contain histidine that renders them naturally resistant to this antibiotic due to a steric hindrance with PHZ molecule. There are many ribosome-targeting antibiotics that, as PHZ (see Table 1), work on one group of bacteria and do not work on the others, however, this phenomenon is usually not referred to as “species-specificity of action” because it is due to the ability of a drug to get inside the bacterial cell rather than a differential activity on its target.

To our knowledge, PHZ is the first example of an antibacterial species-specific ribosome-targeting inhibitor whose activity depends not only on its ability to penetrate inside the cell but also on the fine structure of its target, the bacterial 70S ribosome (regardless of the methylation state of some of the nucleotides in the 23S rRNA). Moreover, PHZ is the second known example of characterized RiPPs from the order *Rhizobiales*. Owing to the ability of nitrogen fixation and establishing symbiotic relationships with leguminous plants, rhizobia are considered as an agriculturally important group of bacteria that have been extensively studied for decades. However, even with dozens of full-genome sequences, the number of rhizobial natural products that were characterized to date remains extremely small in comparison to such fruitful groups as the *Actinobacteria* or the *Enterobacteriales*. At the same time, genome mining reveals significant potential for the discovery of other RiPPs in this group of bacteria. Of particular interest are the genes associated with the biosynthesis of LAPs and Nif11/NHLP-containing peptides^33^ present in multiple rhizobial genomes.

Characterization of PHZ together with the identification of BGCs of its predicted homologs in the genomes of other *Rhizobiales* expands the diversity of LAPs and illustrates how different RiPPs can share similar enzymatic machinery and, at the same time, can exhibit significantly different mechanisms of binding and action upon the target. The narrow-spectrum antibacterial activity of PHZ against rhizobia may serve as a starting point for the development of biocontrol agents, which could be used to combat for example plant-pathogenic *Agrobacterium* strains, or rhizobium inoculants for legume crops that can outcompete endogenous rhizobium strains.

## Methods

### Bacterial strains and growth conditions

The following bacterial strains were used in the present study: *Rhizobium* sp. Pop5, *R. leguminosarum* bv. *viciae* 3841, *R. leguminosarum* bv. *phaseoli* 4292, *R. leguminosarum* bv. *phaseoli* RCR 3622, *R. etli* DSM 11541, *R. tibethicum* DSM 21102, *Agrobacterium tumefaciens* C58C1, *A. rhizogenes* ARQUA1, *Sinorhizobium meliloti* Sm1021, *S. fredii* HH103*, S. medicae* WSM419, *Mesorhizobium ciceri*, *M. loti* MAFF303099, *M. thianshanense* HAMBI 3372, *Azorhizobium caulinodans* ORS571, *E. amylovora, P. ananatis* PA4*, P. putida* KT2440*, Pseudomonas fluorescens* Pf-5*, B. subtilis* 168*, Bacillus cereus* ATCC 4342*, Arthrobacter* sp. ATCC21022, *M. glauca* NRRL B-3735, *Escherichia coli* DH5α*, E. coli* HB101, *E. coli* MRE600, *E. coli* BW25113, and *E. coli* BW25113Δ*tolC*. We used RM medium (per 1 L: 10 g mannitol, 0.5 g K_2_HPO_4_, 0.2 g MgSO_4_, 0.1 g NaCl, 1 g yeast extract, pH 6.8) for cultivation of various species of the *Rhizobium* genus (Table 1). Other rhizobia (including species of the genera *Mesorhizobium*, *Sinorhizobium*, *Agrobacterium*, and *Azorhizobium*) were cultivated in YEB medium (per 1 L: 5 g beef extract, 5 g peptone, 5 g sucrose, 1 g yeast extract, 0.2 g MgSO_4_, pH 7.5) (Table 1). All rhizobia strains were cultivated at 28 °C. Growth of all other strains was performed in LB medium (per 1 L: 5 g NaCl, 10 g tryptone, and 5 g yeast extract) at 28 or 37 °C (for *E. coli* strains). When necessary, antibiotics were used at concentrations of 100 μg/ml for kanamycin (for *S. meliloti* Sm1021), 50 μg/ml for kanamycin (for *E. coli* strains), 25 μg/ml for chloramphenicol, and 500 μg/ml for streptomycin.

### Molecular cloning procedures

Primers used for DNA amplification are listed in Supplementary Table 1. The *rplD* and *rplV* genes were amplified from *Sinorhizobium meliloti* Sm1021 genomic DNA using rplD_pSRK_GA_F/rplD_pSRK_GA_R and rplV_pSRK_GA_F/rplV_pSRK_GA_R pairs of primers, respectively, and cloned into the pSRK (Kan) broad-range host vector^34^ following the Gibson assembly protocol (NEB). Two mutant variants of the *rplD* gene and one mutant variant of *rplV* gene were obtained through PCR-based site-specific mutagenesis with primer pairs rplD_G68H_F/rplD_G68H_R, rplD_K65A_F/rplD_K65A_R, and rplV_K90R_F/rplV_K90R_R, and cloned into the pSRK plasmid as well. The *phzE* gene was amplified from *Rhizobium* sp. Pop5 genomic DNA using phzE_pSRK_GA_F and phzE_pSRK_GA_R primers and cloned into pSRK. The transformation of *Sinorhizobium meliloti* Sm1021 with pRSK and pSRK-derived vectors was performed by triparental mating using pRK600 as a helper plasmid.

### PHZ production and purification

*Rhizobium* sp. Pop5 was cultivated for 24 h in liquid RM medium (750 ml) with shaking (180 rpm) at 28 °C. Next, the cells were pelleted (14,000 rpm, 30 min, 4 °C) and cultivation medium supplied with 0.1% trifluoroacetic acid (TFA) was loaded onto Agilent HF Bond Elut LRC-C18 Cartridge (1 g) pre-equilibrated with 0.1% TFA. The cartridge was then extensively washed with water followed by 50 ml of 10% acetonitrile (MeCN). The peptide fraction was eluted using 10 ml of 30% MeCN, dried out with centrifugal evaporator (GeneVac EZ-2), and dissolved in 500 μl of 100% dimethyl sulfoxide (DMSO). The obtained extract was then subjected to reverse phase HPLC purification on a Luna PREP C18 column (21.2 × 250 mm, 10 μm particle size). The column was pre-equilibrated using 0.1% TFA (Buffer A). The following gradient of 100% MeCN (Buffer B) was used for the separation and elution of the peptides: 0–5 min 0% B, 5–12 min 0–19% B, 12–22 min 19–22% B, 22–27 min 22–70% B, 27–33 70% B. The detection was performed at 254 nm. The collected fractions were then checked with MALDI-ToF MS and those containing the compounds of interest were dried under vacuum, dissolved in 100% DMSO and stored at −20 °C until further use.

### High-resolution MS and ESI-MS-MS analysis

One microgram of peptides in a volume of 1–4 µL was loaded onto the Acclaim µ-Precolumn (0.5 mm × 3 mm, 5 µm particle size, Thermo Scientific) at a flow rate of 10 µL/min for 4 min in an isocratic mode of Mobile Phase C (2% MeCN, 0.1% formic acid). Then the peptides were separated with high-performance liquid chromatography (HPLC, Ultimate 3000 Nano LC System, Thermo Scientific, Rockwell, IL, USA) in a 15-cm long C18 column (Acclaim^®^ PepMap™ RSLC inner diameter of 75 μm, Thermo Fisher Scientific, Rockwell, IL, USA). The peptides were eluted with a gradient of buffer B (80% MeCN, 0.1% formic acid) at a flow rate of 0.3 μl/min. The total run time was 60 min, which included an initial 4 min of column equilibration with buffer A (0.1% formic acid), then gradient from 5% to 35% of buffer B over 35 min, then 6 min to reach 99% of buffer B, flushing 10 min with 99% of buffer B and 5 min re-equilibration with buffer A.

MS analysis was performed at least in triplicate with a Q Exactive HF mass spectrometer (Q Exactive HF Hybrid Quadrupole-OrbitrapTM Mass spectrometer, Thermo Fisher Scientific, Rockwell, IL, USA). The temperature of the capillary was 240 °C and the voltage at the emitter was 2.1 kV. Mass spectra were acquired at a resolution of 120,000 (MS) in a range of 300−1500*m/z*. Tandem mass spectra of fragments were acquired at a resolution of 15,000 (MS/MS) in the range from 100*m/z* to 1200*m*/*z* value determined by a charge state of the precursor, but no more than 2000*m/z*. The maximum integration time was 50 and 110 ms for precursor and fragment ions, respectively. Automatic gain control (AGC) target for precursor and fragment ions were set to 1*10^6^ and 2*10^5^, respectively. An isolation intensity threshold of 50,000 counts was determined for precursor’s selection and up to top 20 precursors were chosen for fragmentation with high-energy collisional dissociation (HCD) at 29 normalized collision energy (NCE). Precursors with a charge state of +1 and more than +5 were rejected and all measured precursors were dynamically excluded from triggering of a subsequent MS/MS for 20 s.

### MALDI-ToF-MS and MS–MS analysis

MALDI-MS mass spectra were recorded in a positive ion measurement mode using Ultraflextreme MALDI-ToF-ToF-MS instrument (Bruker Daltonics, Germany). The spectra were obtained in reflecto-mode with the accuracy of measuring the monoisotopic *m*/*z* ratio up to 0.1 Da. The fragmentation spectra were obtained using the Lift mode, for which the accuracy of measuring the daughter ions was within 0.2 Da. Sample aliquots were mixed on a steel target with a 30 mg/ml 2,5-dihydroxybenzoic acid in 0.5% TFA and a 30% MeCN–water solution (Sigma-Aldrich) (see Supplementary Data 1–4).

### Determination of MICs

Determination of MICs was performed in 96-well plates in different media for different bacterial strains (Table 1) using a microdilution assay. In each row, the next well contained two times less antibiotic than the previous one. Two different concentration ranges were tested: 128–0.5 μM for the strains less susceptible to PHZ; and 8 μM to 31.25 nM for the strains more susceptible to PHZ; a well without antibiotic was used as a control. Initially each well contained 100 μl of media. Actively growing bacteria were added to the wells up to the concentration of ~5*10^6^ cells/mL. The plates were incubated for 48 h at 28 °C with shaking (180 rpm). MIC was defined as the concentration of PHZ in the well, in which no bacterial growth was observed, while the two-fold lower concentration of PHZ was not abolishing the growth of bacteria.

### Testing of PHZ activity in vivo

For the in vivo bioactivity test (Fig. 2a), the reporter strain BW25113-pDualrep2 and BW25113*ΔtolC*-pDualrep2 was used as previously described^19^. Briefly, 2 µL of solutions of tested compounds were applied onto the agar plate that already contained a lawn of the reporter strain. 10 mM PHZ, KLB, ERY, and 20 µM levofloxacin solutions were used. After overnight incubation of the plate at 37 °C, it was scanned by ChemiDoc (Bio-Rad) using “Cy3-blot” settings for RFP and “Cy5-blot” for the Katushka2S protein.

### In vitro translation inhibition assays

The inhibition of firefly luciferase synthesis by PHZ (Fig. 2b) was assessed essentially as described previously^23^. Briefly, the in vitro transcribed firefly luciferase mRNA was translated in the *E. coli* S30 Extract System for Linear Templates (Promega). Reactions containing 200 ng of mRNA and 0.1 mM of d-luciferin were carried out in 5 µL aliquots at 37 °C for 15 min and the activity of in vitro synthesized luciferase was measured by VictorX5 (PerkinElmer) every 30 s.

To test the ability of PHZ to act upon the *E. coli* vs. *T. thermophilus* ribosomes (Supplementary Fig. 3), we used in vitro PURExpress translation system (New England Biolabs) reconstituted from purified components and supplied with either the WT 70S ribosomes from *E. coli* or the reconstituted hybrid 70S ribosomes containing purified 30S subunits from *E. coli* and purified 50S subunits from *T. thermophilus*. Translation of superfolder green fluorescent protein (sfGFP) was carried out according to the manufacturer’s protocol. The assembled reactions (10 μl) were supplemented with 100 ng of sfGFP mRNA. PHZ, KLB, or CHL (chloramphenicol) antibiotics we added to a final concentration of 50 μM when needed. The reactions were placed in a 384-well black-wall plate and the progression of the reactions was monitored over 3 h by a TECAN microplate scanner.

### Toe-printing analysis

Toe-printing analysis was carried out essentially as previously described^35^, using *trpL*-2Ala mRNA as a template for protein translation. *trpL*-2Ala template was prepared by PCR from pDualrep2 plasmid with following oligonucleotides: F TGTAATACGACTCACTATAGGGAATTTTTCTGTATAATAGCCGCGGAAGTTCACGTAAAAAGGGTATCGACAATGAA and R GCGTTAAGGCTATGTACGGGTATCTGATTGCTTTACG, GCGTTAAGGCTATGTAC oligonucleotide was used for reverse transcription. The concentrations of the tested compounds are shown above each lane in Fig. 2e.

### Cryo-EM structure determination

*Ribosome preparation*: *E. coli* strain MRE600 overnight cultures were diluted 1:100 into 3 L of LB. After growth to mid-log phase (OD_600_ = 0.6), cells were cooled on ice for 3 min, pelleted, and washed with buffer A (20 mM Tris–HCl pH 7.5, 100 mM NH_4_Cl, 10 mM MgCl_2_, 0.5 mM EDTA, 2 mM DTT). Cells were resuspended in 100 ml buffer AS (buffer A with 0.15 M sucrose) and lysed with two passes through an Emulsiflex-C3 homogenizer at 18,000 psi. Lysate was clarified by centrifugation for 30 min at 18,000 rpm in a JA-20 rotor (Beckman-Coulter). The supernatant was layered over a sucrose cushion of 24 ml buffer B (buffer A with 500 mM NH_4_Cl) with 0.5 M sucrose and 17 ml buffer C (20 mM Tris–HCl pH 7.5, 60 mM NH_4_Cl, 6 mM MgCl_2_, 0.5 mM EDTA, 2 mM DTT) with 0.7 M sucrose in ti-45 tubes (Beckman-Coulter). Ribosomes were pelleted in a Ti-45 rotor at 27,000 rpm for 16 h at 4 °C. Crude ribosome pellets were resuspended in disassociation buffer (buffer C with 1 mM MgCl_2_) and clarified via centrifugation at 15,000 × *g* for 10 min. The cleared supernatant was layered over 15–40% sucrose gradients in disassociation buffer and spun 28,000 rpm for 16 h at 4 °C in a SW-32 rotor (Beckman-Coulter). Gradients were fractionated with an ISCO fractionation system and 30S + 50S peaks were combined. The combined subunits were concentrated in 15 ml Millipore 100k molecular weight cutoff spin filter and buffer exchanged with reassociation buffer (buffer C with 10 mM MgCl_2_). Ribosomes were incubated 45 min at 37 °C, layered onto 15–40% sucrose gradients in reassociation buffer, and spun 27,000 rpm for 15 h at 4 °C in an SW-32 rotor. Gradients were fractionated as before, 70S peaks were collected, and ribosomes were concentrated and washed with buffer C in an Amicon stirred cell filtration system using 100k cutoff filters. Ribosomes were stored in aliquots at −80 °C.

*Cryo-EM sample preparation*: Roughly 4–6 μM PHZ was incubated with 100 nM ribosomes in RC buffer (50 mM Hepes pH 7.5, 150 mM KOAc, 12 mM MgOAc, 7 mM β-mercaptoethanol) for 30 min. at 37 °C. Complexes were deposited in 4-μl aliquots on 300 mesh Quantifoil UltraAuFoil R1.2/1.3 grids that were topped with a layer of amorphous continuous carbon and glow-discharged for 15 s with a Pelco SC-6 sputter coater. After incubation period of about a minute, the excess sample was washed off on drops of RC–LS buffer (RC with 25 mM KOAc). Samples were blotted and plunge-frozen in liquid ethane using an FEI Vitrobot with blot force 6, humidity 100%, and 20 or 4 °C depending on the freezing session.

*Data collection*: Images were collected in two separate sessions on an FEI Titan Krios Microscope operated at 300 keV with a GIF energy filter and GATAN Summit K2 direct electron detector in super-resolution mode. Images were collected at ×215,000 magnification for a pixel size of 0.56 Å (0.28 Å super-resolution). Automated movie collection was performed with SerialEM^36^ over the defocus range −0.6 to −2.0 μm, and Focus software^37^ was used to monitor data collection. The total dose was 29.97 e^−^/Å^2^ for session 1 and 29.83 e^−^/Å^2^ for session 2, each over 30 frames.

*Data processing:* Cryo-EM data processing was done using RELION 3.0 software^38^ (Supplementary Fig. 9; Supplementary Table 2). The two datasets were processed separately until the final round of map refinement, for which the best particles from both sets were combined. Movies were motion-corrected and dose-weighted using RELION’s motion correction algorithm. Contrast transfer function (CTF) parameters were estimated using CTFFind4^39^ and micrographs with poor CTF fit, as determined by visual inspection, were sorted out. Particles were picked automatically with the Laplacian-of-Gaussian method and subjected to several rounds of careful 2D class-based cleaning before an initial round of 3D auto-refine. CTF refinement with per-particle defocus and beam tilt correction was performed, and particles were further sorted through 3D classification without alignment. Another round of 3D auto-refine was performed on the best 3D classes with focused refinement on the 50S subunit. This was followed by one round of Bayesian polishing^38^ and another round of CTF refinement, which were each followed by another round of 3D auto-refine with a mask around the 50S subunit. Particles from the two sessions were then pooled for the final round of 3D auto-refine. A charge density map was calculated in Chimera^40^ as previously described^41^. Post-processing was also performed in RELION to generate a FSC curve. All refinement procedures were run on 2× binned particles because of the small pixel size.

*Model building*: Assembly #2 from the PDB entry 4YBB^42^ was used as a starting ribosome model. Only the 50S subunit was subjected to real-space refinement in PHENIX^43^, because charge density corresponding to the 30S subunit was relatively poor after focused refinement on the 50S, where PHZ binds. Ions and water molecules were removed from the starting model prior to real-space refinement. The atomic model for the PHZ was built as follows: first, baton building in COOT^44^ was used to trace the main chain; next, the resulting polyalanine chain was mutated to the unmodified PHZ sequence after visual search for the characteristic features in the map; next, oxazole and thiazole modifications were built in Avogadro^45^ version 1.2.0 (http://avogadro.cc) based on the density. Finally, CIF restraints for the oxazole and thiazole moieties were created based on the structural data from ref. ^46^.

All figures showing atomic models were generated using the PyMol software (www.pymol.org). USCF Chimera was used for EM density maps shown in Figs. S5 and S9.

### Reporting summary

Further information on research design is available in the Nature Research Reporting Summary linked to this article.

## Supplementary information

Supplementary Information

Peer Review

Reporting Summary

Description of Additional Supplementary Files

Supplementary Data 1-4

Supplementary Movie 1

## Data Availability

The data that support the findings of this study are available from the corresponding author upon request. Coordinates and charge density map were deposited in the RCSB Protein Data Bank with the accession code 6U48 for the *E. coli* 50S ribosomal subunit in complex with PHZ. The mass-spectrometric data presented in Supplementary Figs. 1C, 2, 3A, B are provided as a Supplementary Data file.
