# Supplementary material for: Structure of ribosome-bound azole-modified peptide phazolicin rationalizes its species-specific mode of bacterial translation inhibition
Source: Nat Commun. 2019 Oct 8;10:4563. doi: 10.1038/s41467-019-12589-5 (PMC6783444; doi:10.1038/s41467-019-12589-5)
Supplement: Supplementary file 1 — Supplementary Information [file 41467_2019_12589_MOESM1_ESM.pdf]

Structure of ribosome-bound azole-modified peptide phazolicin rationalizes its species-specific mode of bacterial translation inhibition

Travin et al.

SUPPLEMENTARY FIGURES

A

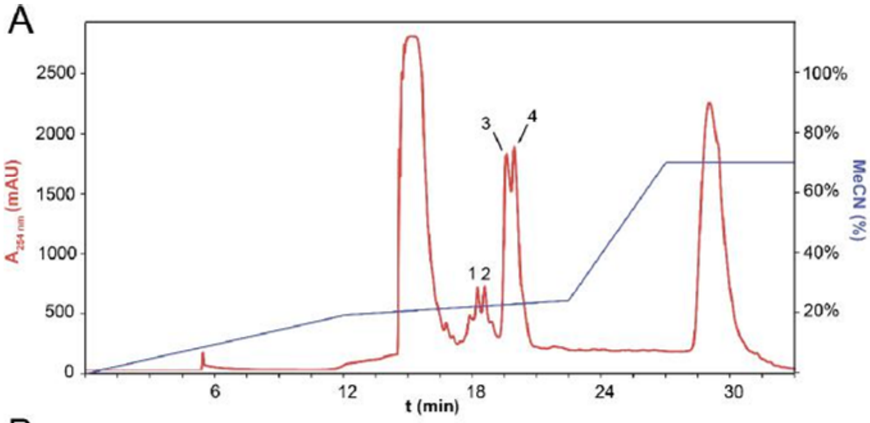

B

	HPLC peak	Formula	Mass (av.)	Number of cycles	Structural features
Major forms	3	ATCARCDSSSRCGASGKSSGSASSIST	2364.5	8	Phasolicin (PHS)
	4	AATCARCDSSSRCGASGKSSGSASSIST	2435.5	8	Alternative leader peptide cleavage site
	4	TAATCARCDSSSRCGASGKSSGSASSIST	2536.6	8	
Minor forms	1	ATCARCDSSSRCGASGKSSGSASSIST	2384.5	7	Smaller number of cycles in the product
	1	ATCARCDSSSRCGASGKSSGSASSIST	2404.5	6	
	1	ATCARCDSSSRCGASGKSSGSASSIST	2424.5	5	
	2	AATCARCDSSSRCGASGKSSGSASSIST	2455.5	7	Alternative leader peptide cleavage site + smaller number of cycles
	2	TAATCARCDSSSRCGASGKSSGSASSIST	2556.6	7	
	1,2	ATCARCDSSSRCGASGKSSGSASS	2083.4	7	C-terminal degradation of the peptide + alternative cleavage
	1,2	AATCARCDSSSRCGASGKSSGSASS	2154.4	7	

C

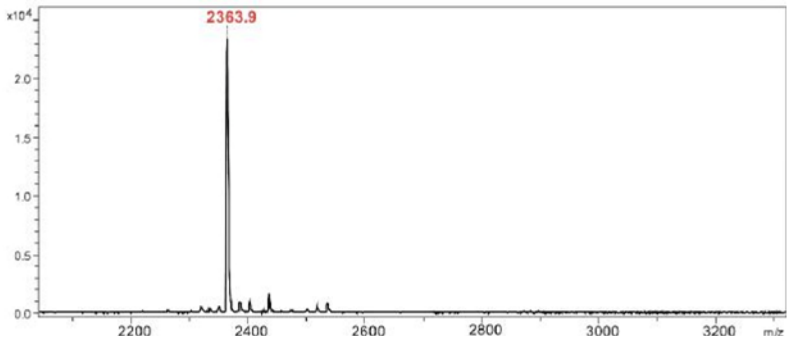

Supplementary Figure 1 | Isolation and purification of PHZ. (**A**) HPLC profile of the crude extract from RM medium after *Rhizobium sp.* Pop5 cultivation. Shown are the 254-nm absorbance curve (red trace) together with the MeCN-gradient profile (blue trace). Note that azole-containing peptides absorb UV at 254-nm wavelength. Each of the HPLC peaks was assigned a number and subsequently analyzed using MS. (**B**) Characteristics of PHZ-related compounds from the medium extracts. Major forms (HPLC peaks 3 and 4) are indicated with the dark blue background. Cyclized residues are highlighted in red in the peptide sequences. (**C**) MS spectra of HPLC peak 3. Molecular mass of 2363.9 $[M+H]^+$ corresponds to PHZ.

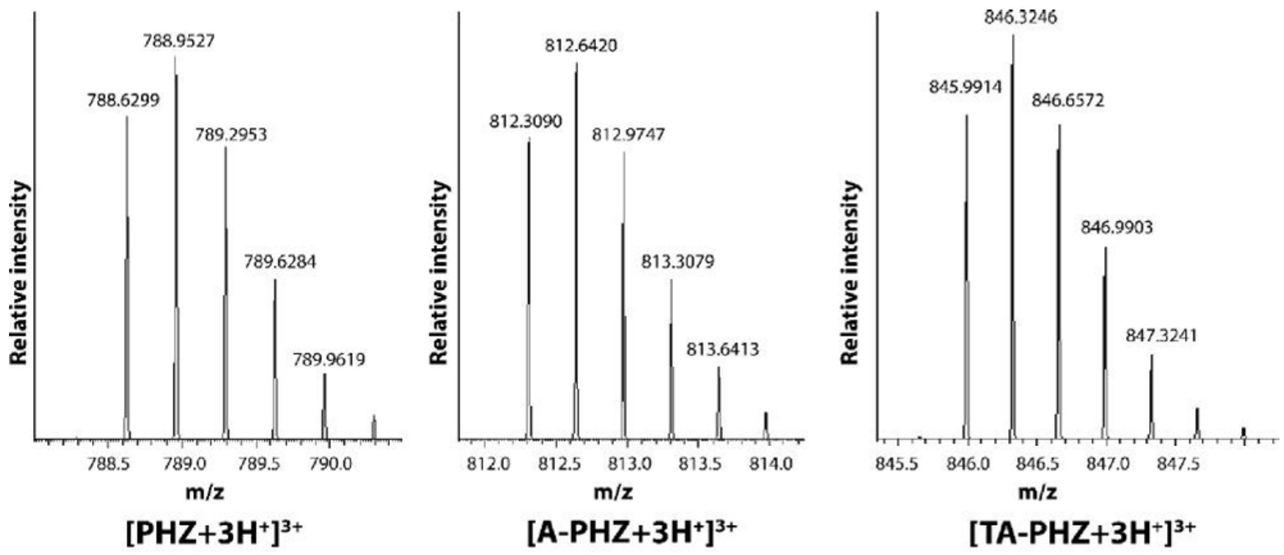

**Calculated MW:**	2362.8651	2433.9022	2534.9499
**Experimental MW:**	2362.8679	2433.9052	2362.9524
**Mass error (ppm):**	1.18	1.23	0.98

Supplementary Figure 2 | High-resolution ESI-MS spectra of the three major compounds related to PhzA precursor peptide identified in the *Rhizobium sp.* Pop5 cultivation medium (mature PHZ and two forms with additional amino acid residues at the N-terminus, resulting from alternative leader cleavage). The m/z values for each peak are indicated. The calculated mass, experimentally obtained mass, and mass error in ppm are shown for each compound.

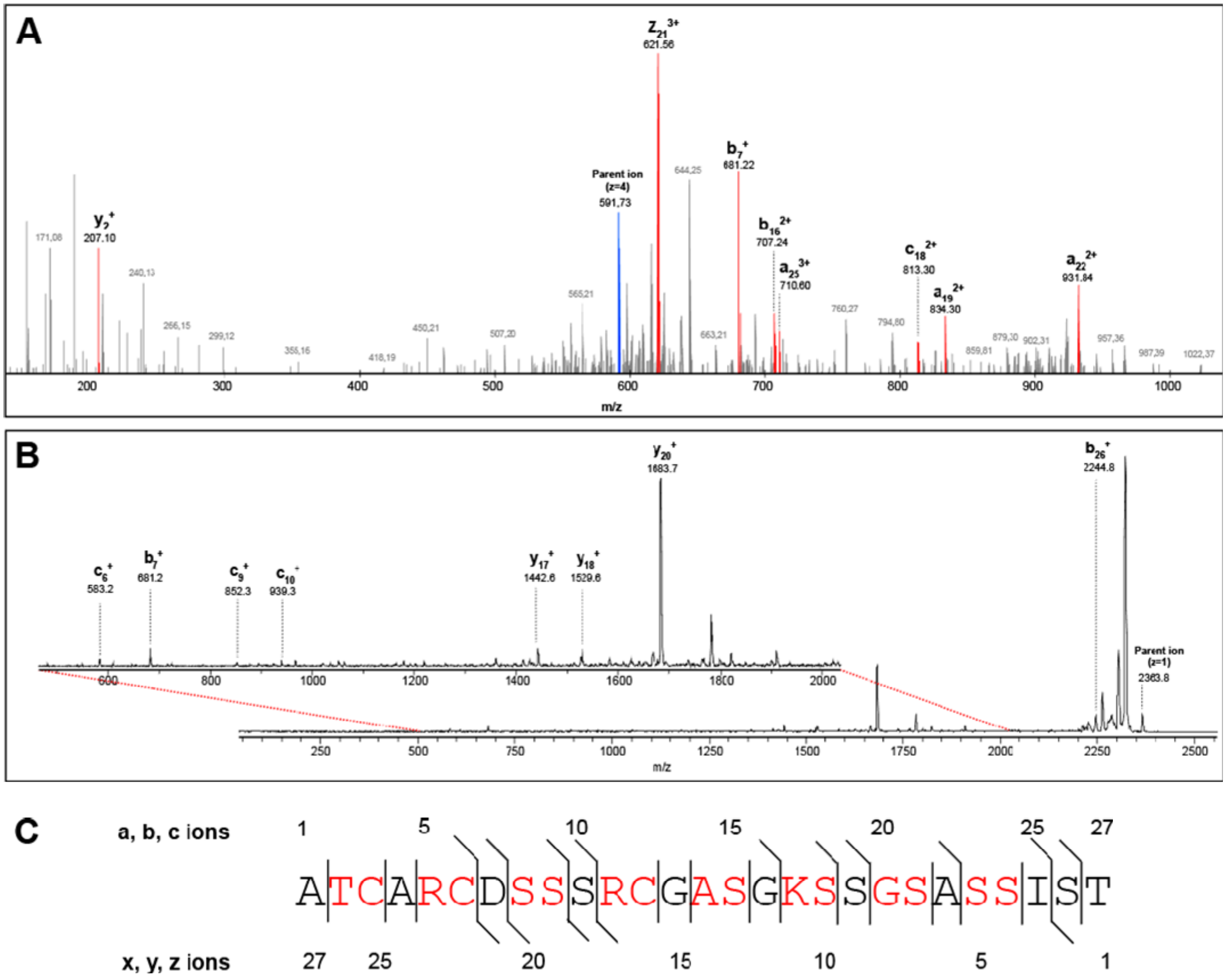

Supplementary Figure 3 | MS/MS analysis of PHZ. (A) ESI-MS/MS spectrum for the ion 591.73 $[M+4H^{+}]^{4+}$. The peaks corresponding to single fragmentation events in the structure of the compound are shown in red and labeled. **(B)** MALDI-MS/MS spectrum for the ion 2363.8 $[M+H^{+}]^{+}$. The range of m/z between 500 and 2050 is shown in detail separately. **(C)** Amino acid sequence of PHZ. The fragments detected in MS/MS analysis are indicated with slashes, residues proposed to be involved in azole cycle formation are red, numeration of ions is shown above (a,b,c series) and below (x, y, z series) the sequence.

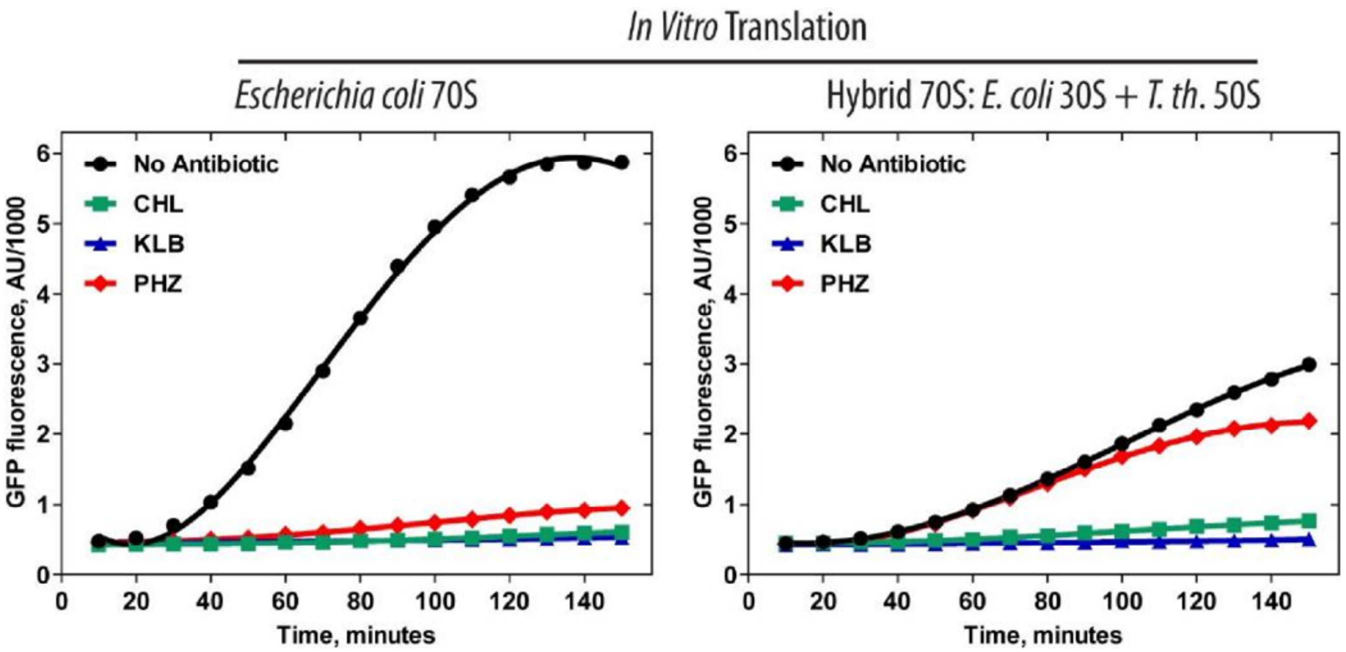

Supplementary Figure 4 | Effects of phazolicin (PHZ), chloramphenicol (CHL), and klebsazolicin (KLB) on *in vitro* synthesis of superfolder green fluorescent protein (sfGFP) using PURExpress system supplied with either the *E. coli* 70S WT ribosomes or hybrid ribosomes (*E. coli* 30S + *Th. thermophilus* 50S). CHL and KLB were used as positive controls. Final concentrations for all antibiotics were 50 μM. Note, that the overall rate of protein synthesis by the hybrid ribosome is ~2-fold lower than that of the WT 70S (black curves).

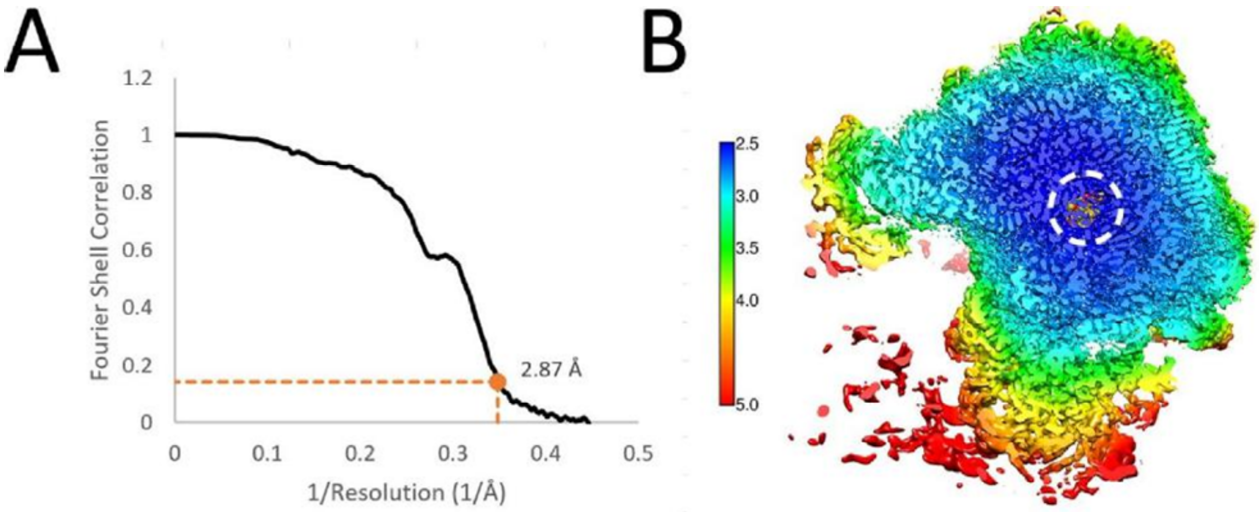

Supplementary Figure 5 | Fourier shell correlation (FSC) curve for the cryo-EM structure of *E. coli* ribosome with PHZ bound. (**A**) Based on the "gold-standard FSC" cutoff value 0.143, the overall resolution for the large ribosomal subunit in the 70S ribosome is 2.87 Å. (**B**) Slice through the 50S subunit showing local resolution limits. Note that the region of PHZ binding (indicated with the dashed white circle) is characterized by the highest local resolution. Color scale bar on the left is in Ångstroms. 30S ribosomal subunit is not shown and was not included in the final model because after the focused refinement on the 50S subunit the local resolution and connectivity of the map for the 30S subunit were poor.

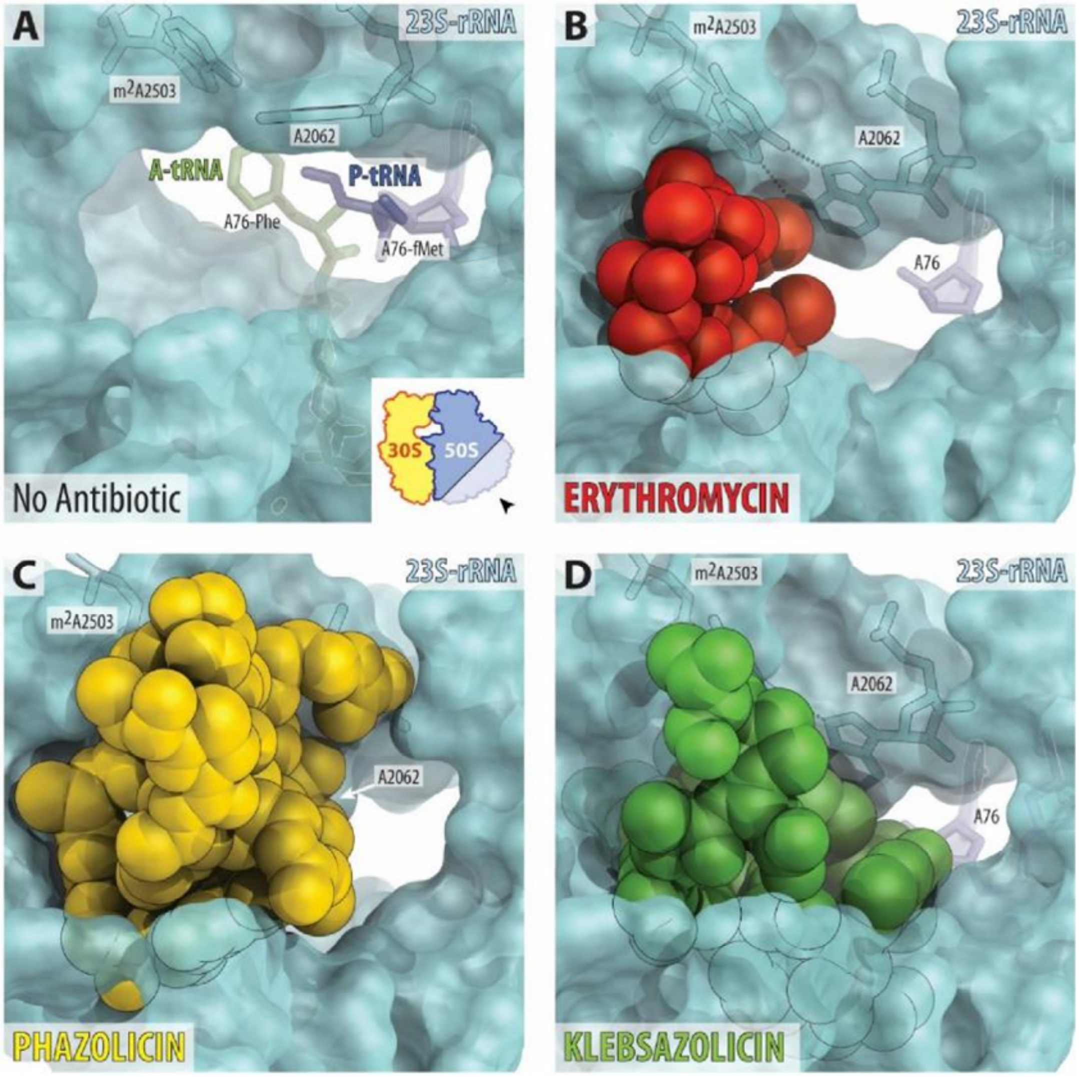

Supplementary Figure 6 | Occlusion of the nascent peptide exit tunnel by antibiotics. (**A**) The lumen of the nascent peptide exit tunnel of the drug-free 70S ribosome (PDB entry 4Y4P[1]). The view is from the wide-open part of the tunnel onto the PTC as indicated by the inset. Nucleotide A76 of the P-site tRNA is shown in dark blue. A-site tRNA is not visible in this view, however, the location of its aminoacylated moiety is shown. Note that nucleotide A2062 of the 23S rRNA is pointed toward the viewer and is not involved in Hoogsteen-edge base pairing with the nucleotide m^2A2503 in the wall of NPET. (**B**, **C**, **D**) Occlusion of the nascent peptide exit tunnel by ERY (B), PHZ (C), and KLB (D). Structures of ERY and KLB are from PDB entries 6ND6[2] and 5W4K[3], respectively. Note that, unlike ERY and similar to KLB, PHZ almost completely occludes the lumen of the peptide exit tunnel. Also note that binding of ERY, PHZ, or KLB causes characteristic rotation of nucleotide A2062 by more than ninety degrees away from the viewer to form Hoogsteen base-pair with the m^2A2503 of the 23S rRNA.

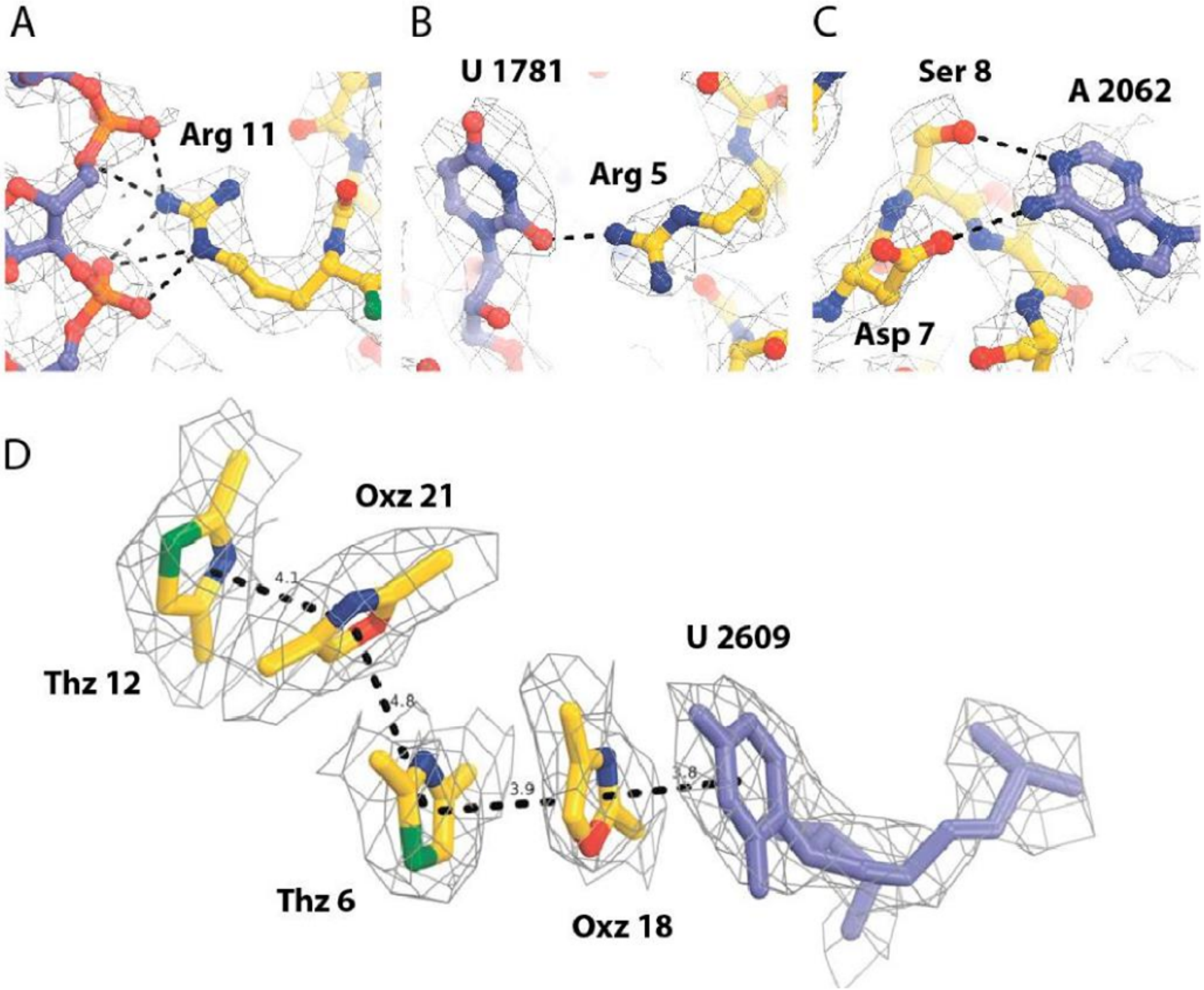

Supplementary Figure 7 | PHZ contacts with the ribosome. (**A-C**) Close-up views of the individual PHZ contacts with the nucleotides of the 23S rRNA. Hydrogen bonds are shown as dashed lines. (**D**) Close-up view of the intramolecular it-it-stacking system and its interaction with the nucleobase U2609 of the 23S rRNA. The directions of face-to-face and edge-to-face it-it stacking interactions are shown as dashed lines. Distances between the planes of aromatic heterocycles are shown in ångstroms (Å).

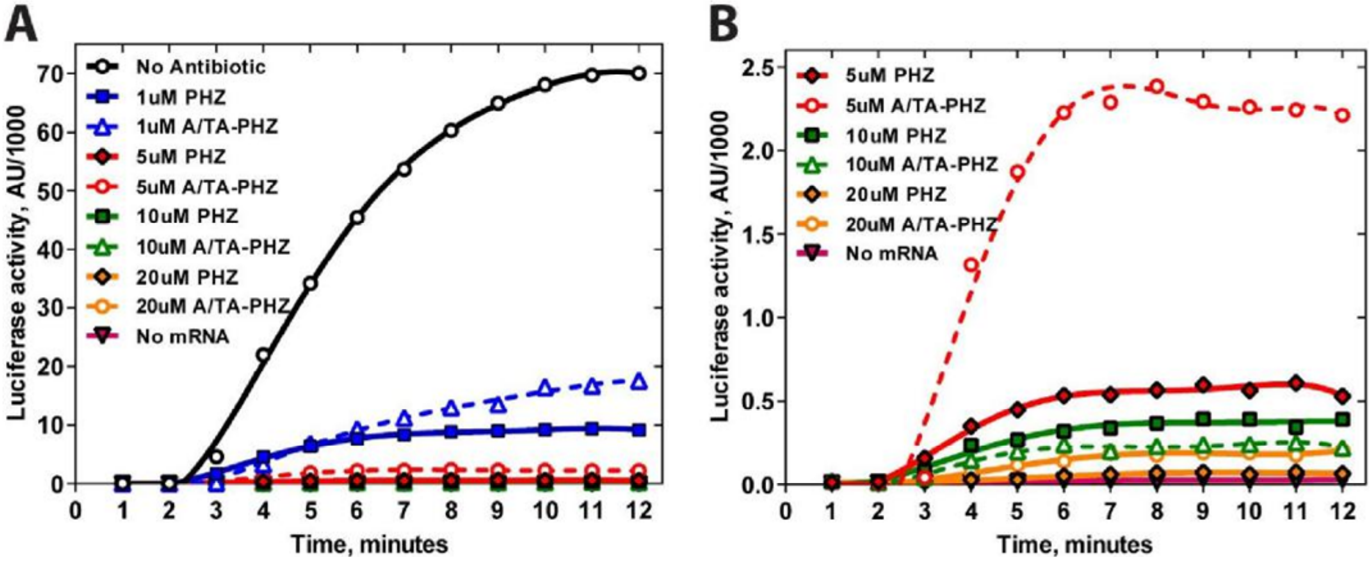

Supplementary Figure 8 | Comparison of the *in vitro* translation inhibition activity of PHZ and its naturally occurring longer forms with additional amino acids at the N-terminus (A-PHZ and TA-PHZ). Graphs corresponding to PHZ are shown as solid lines. Dashed lines correspond to the mixture of long forms of PHZ (A/TA-PHZ, HPLC peak 4, see Fig. S1A). **(B)** is a close-up view of **(A)** in the upper range of antibiotic concentrations (5-20 μM).

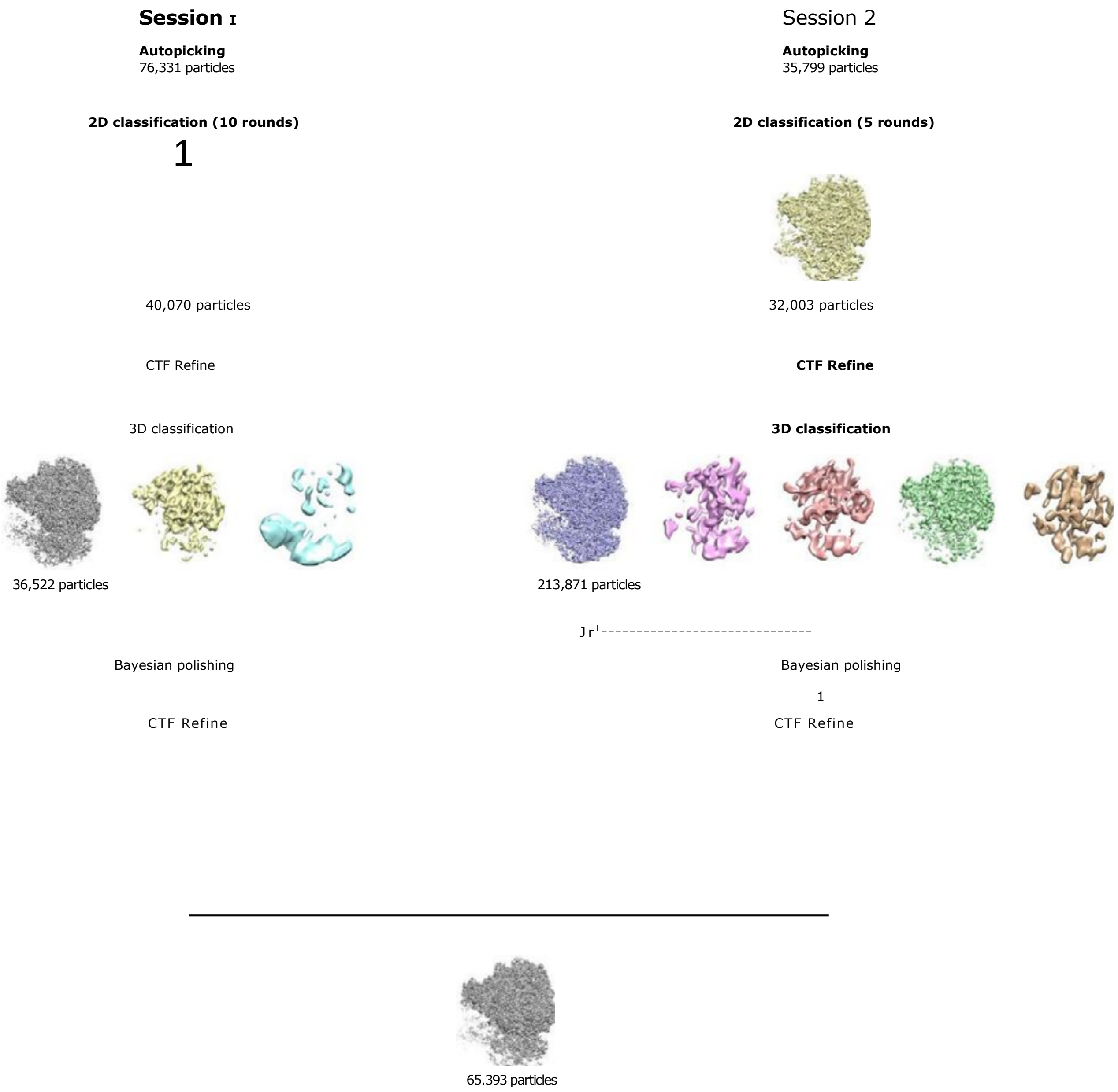

Supplementary Figure 9 | Flowchart showing the steps of cryo-EM data processing in RELION.

SUPPLEMENTARY TABLES

Supplementary Table 1 | Nucleotide sequences of primers used for cloning.

Primer name	Primer sequence	Purpose
rplD_pSRK_GA_F	ATAACAATTTCACACAGGAAACAGCATATGGATCTCACCGTCAAAACCC	Molecular cloning of *rplD* gene into the pSRK plasmid (Gibson Assembly)
rplD_pSRK_GA_R	CGAGGTCGACGGTATCGATATCATTTGAACCGCTCCTCCAGAG	
pSRK_F_GA	ATGCTGTTTCCTGTGTGAAATTG	Amplification of the pSRK plasmid
pSRK_R_GA	TATCGATACCGTCGACCTCG	
rplD_G68H_F	TACAAGCAGAAGCATACGGGCCGCG	Site mutagenesis in *rplD* gene (Gly49His)
rplD_G68H_R	CGCGGCCCGTATGCTTCTGCTTGTACATC	
rplD_K65A_F	GCGCCAAGATGTACGCGCAGAAGGGT	Site mutagenesis in *rplD* gene (Lys65Ala)
rplD_K65A_R	CGTACCCTTCTGCGCGTACATCTTGG	
rplV_pSRK_GA_F	ATAACAATTTCACACAGGAAACAGCATATGGGCAAGGCAAAAGCC	Molecular cloning of *rplV* gene into the pSRK plasmid (Gibson Assembly)
rplV_pSRK_GA_R	CGAGGTCGACGGTATCGATATTATGCGGCCTCCCCTTTG	
rplD_K90R_F	GCTTTTGTTGGCAGGTCGATCGTG	Site mutagenesis in *rplV* gene (Lys90Ala)
rplD_K90R_R	CACGATCGACCTGCCAACAAAAGC	
phzE_pSRK_GA_F	ATAACAATTTCACACAGGAAACAGCATATGGGTAAATCTGAAAGCG	Molecular cloning of *phzE* gene into the pSRK plasmid (Gibson Assembly)
phzE_pSRK_GA_R	CGAGGTCGACGGTATCGATATCATTTGGAAGCAAACG	

Supplementary Table 2 | Cryo-EM data collection, refinement and validation statistics

	50S and PHZ (EMDB-20638) (PDB 6U48)
**Data collection and processing**	
Magnification	215,000x
Voltage (kV)	300
Electron exposure (e–/Å^2)	30
Defocus range (μm)	-0.6 to -2.0
Pixel size (Å)	0.5568 (processed at 1.1136)
Symmetry imposed	none
Initial particle images (no.)	112,130
Final particle images (no.)	65,393
Map resolution (Å)	2.87
FSC threshold	0.143
Map resolution range (Å)	2.5-7
**Refinement**	
Initial model used (PDB code)	4YBB
Model resolution (Å)	2.7
FSC threshold	0.143
Map sharpening *B* factor (Å^2)	n/a*
Model composition	
Non-hydrogen atoms	90950
Protein residues	3356
Ligands	1
B factors (Å^2)	
Protein	33.20
Ligand	14.14
Nucleic Acid	52.39
R.m.s. deviations	
Bond lengths (Å)	0.006
Bond angles (°)	0.946
Validation	
MolProbity score	1.58
Clashscore	3.46
Poor rotamers (%)	0.77
Ramachandran plot	
Favored (%)	93.26
Allowed (%)	6.32
Disallowed (%)	0.43

*Input charge density map for refinement was prepared as described in [4].

SUPPLEMENTARY REFERENCES

1. Polikanov, Y.S., Melnikov, S.V., Soll, D. & Steitz, T.A. Structural insights into the role of rRNA modifications in protein synthesis and ribosome assembly. *Nat. Struct. Mol. Biol.* **22**, 342-344 (2015).

2. Svetlov, M.S. et al. High-resolution crystal structures of ribosome-bound chloramphenicol and erythromycin provide the ultimate basis for their competition. *RNA* **25**, 600-606 (2019).

3. Metelev, M. et al. Klebsazolicin inhibits 70S ribosome by obstructing the peptide exit tunnel. *Nat. Chem. Biol.* **13**, 1129-1136 (2017).

4. Wang, J. Experimental charge density from electron microscopic maps. *Protein Sci.* **26**, 1619-1626 (2017).
