# Supplementary material for: Structure of ribosome-bound azole-modified peptide phazolicin rationalizes its species-specific mode of bacterial translation inhibition
Source: Nat Commun. 2019 Oct 8;10:4563. doi: 10.1038/s41467-019-12589-5 (PMC6783444; doi:10.1038/s41467-019-12589-5)
Supplement: Supplementary file 4 — Description of Additional Supplementary Files [file 41467_2019_12589_MOESM4_ESM.docx]

**Description of Additional Supplementary Files**

File Name: Supplementary Movie 1

Description: PHZ functional site in the bacterial ribosome. The movie shows: (1) zoom-out and (2) close-up views of the PHZ binding site in the large subunit of the *E. coli* ribosome; (3) details of PHZ interactions with the 23S rRNA in the PTC of the ribosome; (4) occlusion of the nascent peptide exit tunnel by the PHZ molecule.

File Name: Supplementary Data 1

Description: High-resolution LC-MS (and tandem MS) spectra for the HPLC fraction containing PHZ used in Supplementary Figure 2 and 3a.

File Name: Supplementary Data 2

Description: High-resolution LC-MS (and tandem MS) spectra for the HPLC fraction containing the forms with alternative leader cleavage site (A-PHZ and TA-PHZ) used in Supplementary Figure 2.

File Name: Supplementary Data 3

Description: MALDI-ToF mass-spectrum of the HPLC fraction containing PHZ used in Supplementary Figure 1c.

File Name: Supplementary Data 4

Description: MALDI-ToF MS-MS spectrum of PHZ (m/z=2363.8 [M+H]^+^) used in Supplementary Figure 3b.
