# Supplementary material for: Structure of ribosome-bound azole-modified peptide phazolicin rationalizes its species-specific mode of bacterial translation inhibition
Source: Nat Commun. 2019 Oct 8;10:4563. doi: 10.1038/s41467-019-12589-5 (PMC6783444; doi:10.1038/s41467-019-12589-5)
Supplement: Supplementary file 2 — Peer Review [file 41467_2019_12589_MOESM2_ESM.pdf]

Reviewers' comments:

Reviewer #1 (Remarks to the Author):

The manuscript by Travin et al. "Phazolicin – A novel thiazole/oxazole-modified peptide inhibiting the bacterial ribosome in a species-specific way" reports the discovery of a new cluster of genes in Rhizobium sp. Pop5 encoding the precursor peptide and modification enzymes required to produce phazolicin (PHZ). PHZ is a heavily modified peptide that exhibits narrow-spectrum antimicrobial activity. It is a linear 27-residue long azol(in)-containing peptide in which serine and cysteine residues become cyclized oxazole and thiazole, respectively. Having a narrow spectrum of activity, crystal structure determination of the Thermus thermophilus ribosome in complex with PHZ did not yield positive difference electron density corresponding to ribosome-bound PHZ, suggesting PHZ has low affinity for T. thermophilus 70S ribosomes. Cell-free translation assay shows that PHZ binds to the large 50S ribosomal subunit of the E. coli ribosome. Thus, to gain insights into the mode of ribosome inhibition by PHZ, the authors used cryogenic electron microscopy (cryo-EM). Their cryo-EM reconstruction of the E. coli 70S ribosome shows that PHZ binds in the nascent peptide exit tunnel of the 50S subunit. The reconstruction shows clear and high quality electron density for ribosome-bound PHZ, allowing the authors to unambiguously trace residues 2-23 in the map. The binding of PHZ to the ribosome shares similarities with klebsazolicin (KLB), another linear peptide containing thiazole and oxazole rings, which the authors have described two years ago. This work expands the repertoire of antimicrobial peptides capable of inhibiting protein translation by binding into the peptide exit tunnel of the ribosome. The work is well executed, clearly presented, and should be published in Nature Communications. This reviewer has a few minor suggestions and comments that should be addressed before publication:

1. In figure 5, the authors are attempting to provide a rationale for the observed species-specificity of PHZ. While the authors explore the effects of mutations of a few residues in ribosomal protein uL4, similar experiments are not performed for uL22. This reviewer is not convinced that Arg90 in uL22 would lead to a clash with PHZ as depicted in Fig. 5C. The flexibility of the arginine side chain may be enough to avoid a clash altogether. What is the identity of residue 90 in S. meliloti? For instance, does expression of uL22 K90R confer resistance to PHZ? The authors should comment on this.

2. In Figure 5C and D, the reader would appreciate to see labels corresponding to N and C termini of PHZ (panel C) and of the C-terminus of KLB (panel D).

3. In the discussion section, the authors make the point that PHZ is the first example of a ribosome-targeting antibiotic whose binding is affected by the fine structure of the ribosome, making it species-specific. The authors should revise the discussion and incorporate structural data from the Steitz group who showed that the identity of nucleotide 2058 in the peptide exit tunnel determines the sensitivity to erythromycin (Tu D, et al. Cell 2005). For example, archeon and eukaryotes are naturally resistant to the antibiotic erythromycin because position 2058 is a G, while in eubacteria, it is A. Adenosine forms favorable interactions with erythromycin, while G does not. Thus, this macrolide displays species-specific mode of interactions.

4. In figure 1A, the "violet" color is hard to see. It is too dark and appears almost black.

5. On page 10, line 2, "…the size of PHZ-induced inhibition zones between…" My understanding is that this is a color-based assay, and not a growth inhibition experiment.

6. In same paragraph, "…comparable in size between the two tolC- and wild-type…" would be clearer to use ΔtolC as in the figure, as the superscripted minus sign is too small.

7. On page 14, last sentence on the page, needs re-phrasing. Also "rich" is misspelled and should be "reach"

8. Figure 4, for the reader that is not too familiar with the “toe-printing” assay, why does the ribosome stop at odd codon positions 1, 3, 5, 7…? One or two sentences in figure legend should address this issue.

Reviewer #2 (Remarks to the Author):

Travin and colleagues describe the mechanism of action of phazolicin, a peptide inhibitor of bacterial translation. The authors find that phazolicin inhibits growth of Rhizobium, Sinorhizobium and some other bacteria, including E. coli, but is less efficient against Agrobacterium, Mesorhizobium and plant- or soil-associated bacteria. To understand the narrow-spectrum specificity of this peptide antibacterial, the authors demonstrate that phazolicin inhibits translation in the E. coli cell extract. Toe-printing analyses reveal that the antibiotic stalls translation elongation. Next, the authors describe a cryo-EM structure of the compound bound to E. coli 70S ribosome. Although the 30S subunit is poorly resolved, the compound’s binding site at the core of the 50S subunit is resolved at sub-3A resolution, allowing detailed interpretation of the interactions of phazolicin with ribosomal residues. The authors propose that a site of interaction with protein L4 is critical for species specificity. Indeed, they demonstrate that a T. thermophilus-like L4 mutation in this structure region confers bacterial resistance to phazolicin, also rationalizing the authors’ inability to co-crystallize T. thermophilus ribosomes with phazolicin.

In summary, this is a well-designed study, which reveals the detailed biochemical mechanism of a novel species-specific antibacterial. The manuscript is well written, illustrations are clear and the conclusions are supported by experimental evidence.

The following minor points should be addressed/corrected prior to publication:

1. In Discussion, the following phrase should be corrected: “…which apparently improves the rigidity and stability…”. It is unclear what the “improvement” is relative to. The authors could use a verb that does not imply comparison, e.g. “which apparently confers the rigidity and stability”.

2. In Discussion, the first sentence of the second paragraph is confusing and should be rewritten. Some instances of “its” appear to refer to different nouns. Does “its methylation” refer to the methylation of the 70S ribosome or the inhibitor?

3. A table with structure refinement and validation statistics (e.g. correlation coefficients, RMS bonds/angles, Ramachandran outliers…) is missing.

*****************************************************************************************

# Response to Reviewer #1

*****************************************************************************************

***Comments for the Authors:***

*The manuscript by Travin et al. "Phazolicin – A novel thiazole/oxazole-modified peptide inhibiting the bacterial ribosome in a species-specific way" reports the discovery of a new cluster of genes in Rhizobium sp. Pop5 encoding the precursor peptide and modification enzymes required to produce phazolicin (PHZ). PHZ is a heavily modified peptide that exhibits narrow-spectrum antimicrobial activity. It is a linear 27-residue long azol(in)-containing peptide in which serine and cysteine residues become cyclized oxazole and thiazole, respectively. Having a narrow spectrum of activity, crystal structure determination of the Thermus thermophilus ribosome in complex with PHZ did not yield positive difference electron density corresponding to ribosome-bound PHZ, suggesting PHZ has low affinity for T. thermophilus 70S ribosomes. Cell-free translation assay shows that PHZ binds to the large 50S ribosomal subunit of the E. coli ribosome. Thus, to gain insights into the mode of ribosome inhibition by PHZ, the authors used cryogenic electron microscopy (cryo-EM). Their cryo-EM reconstruction of the E. coli 70S ribosome shows that PHZ binds in the nascent peptide exit tunnel of the 50S subunit. The reconstruction shows clear and high-quality electron density for ribosome-bound PHZ, allowing the authors to unambiguously trace residues 2-23 in the map. The binding of PHZ to the ribosome shares similarities with klebsazolicin (KLB), another linear peptide containing thiazole and oxazole rings, which the authors have described two years ago. This work expands the repertoire of antimicrobial peptides capable of inhibiting protein translation by binding into the peptide exit tunnel of the ribosome. The work is well executed, clearly presented, and should be published in Nature Communications. This reviewer has a few minor suggestions and comments that should be addressed before publication:*

1. *In figure 5, the authors are attempting to provide a rationale for the observed species-specificity of PHZ. While the authors explore the effects of mutations of a few residues in ribosomal protein uL4, similar experiments are not performed for uL22. This reviewer is not convinced that Arg90 in uL22 would lead to a clash with PHZ as depicted in Fig. 5C. The flexibility of the arginine side chain may be enough to avoid a clash altogether. What is the identity of residue 90 in S. meliloti? For instance, does expression of uL22 K90R confer resistance to PHZ? The authors should comment on this.*

   <u>Response:</u> **Excellent point! We are thankful to the reviewer for bringing this up because we actually thought too about doing exactly this experiment, and while the initial version of this manuscript was under review we have obtained the new data. To address the reviewer's comment we have cloned the rplV gene of *Sinorhizobium meliloti* (encoding for uL22 protein) into the pSRK vector and introduced a single amino acid substitution K90R in it (both *S. meliloti* and *E. coli* uL22 have a Lys residue at this position, while *T. thermophilus* carries an Arg residue in the equivalent position). We found that, exactly as suggested by the reviewer, there was no resistance to PHZ in the cells over-producing RplV-K90R mutant, perhaps due to the availability of extra space adjacent to this residue in the ribosome and/or due to the flexibility of arginine residue. The new data are shown in the revised version of Figure 5F. Also, the "Methods" section describing the generation of this new mutant strain and the relevant parts of the "Results" section has been modified accordingly.**

2. *In Figure 5C and D, the reader would appreciate to see labels corresponding to N and C termini of PHZ (panel C) and of the C-terminus of KLB (panel D).*

**<u>Response:</u> Following the reviewer's suggestion, we have added labels pointing to the termini of both PHZ and KLB peptides (see the revised Figure 5).**

3. *In the discussion section, the authors make the point that PHZ is the first example of a ribosome-targeting antibiotic whose binding is affected by the fine structure of the ribosome, making it species-specific. The authors should revise the discussion and incorporate structural data from the Steitz group who showed that the identity of nucleotide 2058 in the peptide exit tunnel determines the sensitivity to erythromycin (Tu D, et al. Cell 2005). For example, archeon and eukaryotes are naturally resistant to the antibiotic erythromycin because position 2058 is a G, while in eubacteria, it is A. Adenosine forms favorable interactions with erythromycin, while G does not. Thus, this macrolide displays species-specific mode of interactions.*

**<u>Response:</u> Indeed, the reviewer is right and by this comment pointed out that some of our statements are not carefully phrased. We wholeheartedly agree with the reviewer that PHZ is not the only example exhibiting species-specific mechanism of on-target action and the suggested example with erythromycin binding to bacterial and not binding to archaeal/eukaryotic ribosomes is a good illustration. There are actually many other such examples and, in fact, absolutely all antibiotics (including ribosome-targeting ones) have species-specificity of action – this is an inherent feature of the selectivity principle that an antibiotic kills some organisms (bacteria) but does not kill the others (eukaryotes). Perhaps, the reviewer misunderstood us here. What we've actually meant is that PHZ exhibits species-specificity among bacteria, not all species in general. And this antibacterial species-specificity is defined by the fine structure of the ribosome but not by the ability of the drug to get inside the cell as in the case with most other ribosome-targeting antibiotics. For example, erythromycin does not work against all bacterial species equally – it is very active against many Gram-positive bacteria and barely active against Gram-negative species. However, if we purify ribosomes from both Gram(+) and Gram(-) bacteria and compare binding affinities or *in vitro* inhibitory properties of erythromycin – they will be nearly the same. In other words, if erythromycin reaches its target (the ribosome), it doesn't matter from with bacteria was that ribosome. However, for PHZ to be active the target ribosome must not have histidine residue in the uL4 protein, which immediately excludes those bacteria that have such histidine. Taking these considerations into account, we would like to respectfully refrain from discussing the reviewer-suggested erythromycin case because, in our opinion, it is irrelevant, although so dear to our hearts.**

**To further strengthen and clarify the above points, we have revised the first sentence of the second paragraph in the Discussion section to read "*To our knowledge, PHZ is the first example of an antibacterial species-specific ribosome-targeting inhibitor whose activity depends not only on its ability to penetrate inside the cell but also on the fine structure of its target, the bacterial 70S ribosome (regardless of the methylation state of some of the nucleotides in the 23S rRNA)*". In the revised version of this sentence, we have emphasized that species-specificity mentioned above refers to bacterial species (and not all species in general) and results not from differential penetration into the cell but rather from the ability to bind to the ribosome. Also, please refer to our response to comment #2 of the second reviewer.**

4. *In figure 1A, the "violet" color is hard to see. It is too dark and appears almost black.*

**<u>Response:</u> Following the reviewer's suggestion, we have changed the hard-to-see violet color to green (which should be easier to see) in the revised version of Figure 1A.**

5. *On page 10, line 2, "…the size of PHZ-induced inhibition zones between…" My understanding is that this is a color-based assay, and not a growth inhibition experiment.*

   **Response: It appears to be a misunderstanding here because in our double-reporter assay we observed expression of fluorescent proteins (either RFP or Katushka2S) in the cells located at the boundary between the bona fide inhibition zone and the lawn of unaffected/uninhibited cells. These cells (located at the boundary) are exposed to sublethal concentrations of antimicrobial agents and, therefore, are alive but can respond to these agents by altering the expression levels of the reporters. Thus, in addition to making inferences about the mechanism of cell growth inhibition (by recording an elevated expression of either RFP or Katushka2S at the boundary of growth inhibition zone), we can also determine the sizes of growth inhibition zones.**

6. *In same paragraph, "…comparable in size between the two tolC- and wild-type…" would be clearer to use ΔtolC as in the figure, as the superscripted minus sign is too small.*

   **Response: Corrected as suggested by the reviewer.**

7. *On page 14, last sentence on the page, needs re-phrasing. Also "rich" is misspelled and should be "reach".*

   **Response: We are thankful to the reviewer for catching this glitch and have re-phrased the sentence.**

8. *Figure 4, for the reader that is not too familiar with the "toe-printing" assay, why does the ribosome stop at odd codon positions 1, 3, 5, 7…? One or two sentences in figure legend should address this issue.*

   **Response: The observed stalling of the ribosome noticed by the reviewer is related to the formation of the secondary structures by the specific mRNA that we used as a template in our toe-printing assay. This stalling is seen in the absence of any inhibitors in the reaction and is thus not relevant. This is now mentined in the text. The relevant toe-prints are the ones that appear in the presence of PHZ in a concentration-depedent manner and they are discussed in the text.**

***************************************************************************************

# Response to Reviewer #2

***************************************************************************************

***Comments for the Authors:***

*Travin and colleagues describe the mechanism of action of phazolicin, a peptide inhibitor of bacterial translation. The authors find that phazolicin inhibits growth of Rhizobium, Sinorhizobium and some other bacteria, including E. coli, but is less efficient against Agrobacterium, Mesorhizobium and plant- or soil-associated bacteria. To understand the narrow-spectrum specificity of this peptide antibacterial, the authors demonstrate that phazolicin inhibits translation in the E. coli cell extract. Toe-printing analyses reveal that the antibiotic stalls translation elongation. Next, the authors describe a cryo-EM structure of the compound bound to E. coli 70S ribosome. Although the 30S subunit is poorly resolved, the compound's binding site at the core of the 50S subunit is resolved at sub-3A resolution, allowing detailed interpretation of the interactions of phazolicin with ribosomal residues. The authors propose that a site of interaction with protein L4 is critical for species specificity. Indeed, they demonstrate that a T. thermophilus-like L4 mutation in this structure region confers bacterial resistance to phazolicin, also rationalizing the authors' inability to co-crystallize T. thermophilus ribosomes with phazolicin.*

*In summary, this is a well-designed study, which reveals the detailed biochemical mechanism of a novel species-specific antibacterial. The manuscript is well written, illustrations are clear and the conclusions are supported by experimental evidence.*

*The following minor points should be addressed/corrected prior to publication:*

1. *In Discussion, the following phrase should be corrected: "…which apparently improves the rigidity and stability…". It is unclear what the "improvement" is relative to. The authors could use a verb that does not imply comparison, e.g. "which apparently confers the rigidity and stability".*

   **Response:** **Following the reviewer's suggestion, we have modified the corresponding sentence to read "*Unlike KLB, which does not appear to have extensive intramolecular interactions, four out of eight azole rings of PHZ form continuous π-π-stacking system (Figure S5D), which apparently confers the rigidity and stability of the antibiotic in its binding site*".**

2. *In Discussion, the first sentence of the second paragraph is confusing and should be rewritten. Some instances of "its" appear to refer to different nouns. Does "its methylation" refer to the methylation of the 70S ribosome or the inhibitor?*

   **Response:** **We completely agree with the reviewer that indeed it is unclear from the last portion of the sentence whether "*its*" refers to the ribosome or the drug. To remove any ambiguity, we have revised this sentence to read "*To our knowledge, PHZ is the first example of an antibacterial species-specific ribosome-targeting inhibitor whose activity depends not only on its ability to penetrate inside the cell but also on the fine structure of its target, the bacterial 70S ribosome (regardless of the methylation state of some of the nucleotides in the 23S rRNA)*". In the revised version of this sentence, we have emphasized that aforementioned species-specificity refers to bacterial species (and not all species in general) and results not from differential penetration into the cell but rather from the ability to bind to the ribosome. Also, please refer to our response to comment #3 of the first reviewer.**

3. *A table with structure refinement and validation statistics (e.g. correlation coefficients, RMS bonds/angles, Ramachandran outliers…) is missing.*

**<u>Response:</u>** **This table was actually included in the "Supplementary Information" section as per *Nature Communications* requirements. This table has been slightly revised and still appears as Table S3 in the "Supplementary Information" section.**

REVIEWERS' COMMENTS:

Reviewer #1 (Remarks to the Author):

The authors have carefully addressed this reviewer's comments and concerns. In my view, the manuscript is ready for publication.
